# Gastric Mixing and Acid Diffusion in a Human Stomach Simulated Using Smoothed Particle Hydrodynamics

**DOI:** 10.1111/1750-3841.70671

**Published:** 2025-11-07

**Authors:** Xinying Liu, Simon M. Harrison, Shouryadipta Ghosh, David F. Fletcher, Paul W. Cleary

**Affiliations:** ^1^ School of Chemical and Biomolecular Engineering The University of Sydney Sydney NSW Australia; ^2^ CSIRO Data61 Clayton South VIC Australia

**Keywords:** acid diffusion, digestion, gastric mixing, smoothed particle hydrodynamics, stomach

## Abstract

This study develops a computational stomach model using the smoothed particle hydrodynamics (SPH) approach to investigate the effects of fluid viscosity, gastric motility, and acid diffusion rate on gastric mixing and acid distribution. The model incorporates antral contraction waves (ACW) and acid diffusion from the stomach wall. Our findings reveal that the mixing pattern generated by ACWs is strongly anisotropic. When the ACWs travel along the wall, highly effective tangential flow moves alongside and drives the circulatory flow and mixing. Conversely, the weak radial advection orthogonal to the wall provides minimal enhancement to the predominantly radial diffusion. Fluid viscosity influences the mixing dynamics within the stomach significantly, with higher viscosity fluids exhibiting slower and less efficient mixing. Gastric motility, characterized by ACW speed and occlusion, has a substantial impact on enhancing gastric mixing. Increased ACW speed and greater occlusion effectively promote more rapid mixing of the stomach contents. The diffusion coefficient emerges as the dominant factor in acid distribution, overtaking the impact of physical mixing induced by ACWs. This indicates that while mechanical mixing is crucial for overall fluid dynamics, acid diffusion has a greater impact on the distribution of acid and changes in pH within the stomach. These insights emphasize the complex interaction between mechanical and chemical processes in the stomach, highlighting the importance of considering both factors in developing accurate models for gastric digestion and nutrient absorption.

## 1 Introduction

Digestive processes in the stomach can be divided into two main steps: mechanical and chemical breakdown (Bornhorst and Singh 2014). Mechanical breakdown involves processes such as crushing, fracturing, and squeezing and is caused by the deformation of the food arising from interaction with the contracting stomach wall and the applied fluid stresses (Bornhorst et al. 2016). Chemical breakdown involves the secretion of gastric acid from the stomach wall and the interaction between gastric acid and gastric contents (Kong and Singh 2008). These two processes are interconnected. For instance, the mechanical breakdown of a food fragment will expose a fresh contact area for gastric acid to enhance chemical digestion. This can lead to the softening of the food structure, which enables easier mechanical breakdown (Kong and Singh 2008). Understanding human digestion offers the opportunity for new and more effective and/or targeted treatments for gastric diseases, as well as advice regarding diet selection, drug delivery, and the development of functional food product.

Researchers have been trying to understand the complexities of gastric function through various methodologies, including in vivo, in vitro, and in silico approaches. Traditionally, in vivo (within the living organism) and in vitro (outside the living organism, in a controlled environment) methods have dominated the field due to their direct and observable nature. However, with the rapid advancements in computing power and algorithm development, in silico (computational) approaches have emerged as a viable and powerful option. As a result, in silico approaches are being increasingly employed to enhance comprehension of gastric function by incorporating diverse elements of gastric geometry, motility, chemistry, and food composition (Liu et al. 2024).

The primary function of the stomach wall, which drives both mechanical and chemical digestion, is the ACW. The first in silico stomach model, developed by Pal et al. (2004), investigated the dynamics of the ACW through magnetic resonance imaging (MRI) studies. These dynamics have since been incorporated into most of the developed in silico stomach models (Ferrua and Singh 2010, 2011; Kozu et al. 2010; Imai et al. 2013; Miyagawa et al. 2016; Harrison et al. 2018; Ishida et al. 2019; Kuhar et al. 2022). Kozu et al. (2010) found that both the viscosity of the gastric content and the characteristics of the ACWs had a significant impact on the flow phenomena. Alokaily et al. (2019) performed a parametric study by varying the fluid viscosity, speed, width, and maximum relative occlusion of the ACW. It was found that the mixing efficiency improved with an increase in relative occlusion, and a decrease in viscosity or wave width. However, both studies only simulated a very simplified 2D model of the antrum and not the entire stomach.

Acid secretion is the other major function of the stomach wall, which is essential for the chemical breakdown process. Only a few studies have incorporated acid secretion into their models along with the ACW (Lee et al. 2022; Li and Jin 2021; Li et al. 2021; Li and Jin 2023). Lee et al. (2022) studied the effect of gastric motility (including posture, speed, and relative occlusion of the ACW) on drug dissolution with quantified emptying rates. However, the simulated acid was not secreted from the stomach wall but from the dissolved drug. Li and Jin (2021) simulated gastric secretion originating from the stomach's upper region and monitored the corresponding pH fluctuations within the gastric contents. In their subsequent studies, they used the same model to simulate the mixing and emptying of gastric contents with different species (Li et al. 2021), as well as the digestion of meat proteins (Li and Jin 2023). However, their study did not examine the effect of gastric motility.

Most developed stomach models have utilized the traditional mesh‐based finite volume method (FVM) due to its advanced development and ability to provide accurate solutions (Liu et al. 2024). However, FVM lacks robustness when implementing gastric motility and food disintegration compared with particle‐based methods, such as smoothed particle hydrodynamics (SPH), or hybrid approaches like lattice Boltzmann and immersed boundary methods. The particle‐based SPH method has been validated in various applications, including die casting (Cleary et al. 2014), mixing tank flow (Prakash et al. 2007), and peristaltic flow (Liu et al. 2023a).

SPH is well‐suited to simulate gastric mixing and emptying when the moving stomach wall is involved, as demonstrated in Harrison et al. (2018), who developed a biomechanical‐SPH model to investigate fluid mixing and emptying with the ACW dynamics. In contrast, FVM would require remeshing to accommodate large wall deformations, whereas SPH handles such deformations without remeshing. Liu et al. (2023b) examined the buoyancy effect on fluid flow using a static stomach geometry in both FVM and SPH frameworks. The Rayleigh–Taylor instability was well–captured in the SPH model but less evident in the FVM model, suggesting that SPH is better suited for handling flows with interfacial instabilities and when multiple food layers are present. Moreover, recently, Ghosh et al. (2025) demonstrated the ability of SPH to include solid phase materials to investigate the mechanical breakdown and fragmentation of solid foods in a deforming stomach. The ability of SPH in handling complex geometry, deforming walls, free surfaces, interfacial instabilities, and included solids and their fragmentation highlights its promise as a comprehensive method for stomach modeling.

In this study, acid diffusion is incorporated into the previous model developed by Harrison et al. (2018), with the ACW dynamics represented as functions of time and spatial position and applied to the stomach boundary. The aim is to investigate the relationship between the acid transport process and fluid mixing behavior in a deforming stomach, to better understand the mechanisms of physical and chemical digestion processes. The study examines the effects of several parameters, including fluid viscosity, the speed and relative occlusion of the ACW, and the acid diffusion coefficient. Their impacts on acidity levels and mixing efficiency are analyzed and discussed.

## 2 Methods

### 2.1 Numerical Methods

In the SPH method, the motion of fluid particles is described by ordinary differential equations. Detailed descriptions of the method are available in Monaghan (1994), Monaghan (2005), Cleary et al. (2007), and Cleary et al. (2021). The model was built using the CSIRO SPH code developed by Cleary (1998).

#### 2.1.1 Fluid Flow

In SPH, values of physical quantities such as density and velocity at a particular point are computed based on the contributions from the surrounding particles. The interpolated value of a function *F* at a position *r*, is obtained by summing over all particles located within a distance related to the smoothing length *h* from point *r*, as described by Monaghan (1994):

(1) $$F\;\left(r\right)=\sum_{b}m_{b}\;\frac{F_{b}}{\rho_{b}}W\left(r-r_{b},h\right)$$

where $F_{b}$ represents the value of function F at position $r_{b}$, $m_{b}$, and $\rho_{b}$ are the mass and density of fluid particle b, and W denotes the interpolation kernel function with smoothing length h, evaluated based on the distance $|r-r_{b}|$ from the point of interest. Following Monaghan (2005), the smoothing length h is chosen as 1.2 times the initial particle spacing, Δx.

The conservation equation for mass can be formulated as (Monaghan 1992; Monaghan 1994):

(2) $$\frac{d\rho_{a}}{dt}=\sum_{b}m_{b}\;v_{ab}\cdot \nabla_{a}W_{ab}$$

where $\rho_{a}$ is the density of fluid particle a, t denotes time, $v_{ab}=v_{a}\;-v_{b}$, defines the relative velocity between fluid particles a and b, while W is the interpolation kernel function, evaluated using h at a distance $|r-r_{b}|$ from the reference point. The cubic‐spline kernel is used for W as in Harrison et al. (2018) and Liu et al. (2023b).

Fluid pressure is computed from particle density using a weakly compressible approach. This technique involves applying an equation of state, expressed as:

(3) $$P=P_{0}\;\left[{\left(\frac{\rho }{\rho_{0}}\right)}^{\gamma }-1\right]$$

where P denotes fluid pressure; $P_{0}$ is the pressure scaling coefficient; ρ represents the density of the fluid particle; $\rho_{0}$ refers to the reference density (assigned to be 1000 kg/m^3^ for an aqueous liquid in this study).

The pressure scale factor $P_{0}$ used in Equation (3) is determined by

(4) $$\frac{\gamma P_{0}}{\rho_{0}}={c_{s}}^{2}={\left(10U\right)}^{2}$$

where γ (defined as 7) is a material constant applicable to fluids with water like properties (Courant 1944), and $c_{s}$ is the local speed of sound. To ensure weak compressibility, $c_{s}$ must be at least 10 times greater than the characteristic fluid velocity (U), yielding a Mach number of no more than 0.1. This constraint maintains density variation within each fluid below 1%, thereby producing effectively incompressible flow behaviour (Monaghan 1994).

The governing conservation equation for momentum is expressed as:

(5) $$\begin{array}{ccc} \frac{dv_{a}}{dt} & = & -\sum_{b}m_{b}\left[\left(\frac{P_{b}}{\rho_{b}^{2}}+\frac{P_{a}}{\rho_{a}^{2}}\right)-\frac{\kappa }{\rho_{a}\rho_{b}}\frac{4\mu_{a}\mu_{b}}{\left(\mu_{a\;}+\mu_{b}\right)}\frac{v_{ab}\times r_{ab}}{\left(r_{ab\;}^{2}+\eta^{2}\right)}\right]\\ & & \cdot \nabla_{a}W_{ab}+\sum_{k}f_{ak}+g \end{array}$$

where $P_{a}$ and $P_{b}$ represent the pressures of fluid particles a and b, $\mu_{a}$ is the viscosity of particle a, κ is a calibration constant associated with the viscous term, as described by Cleary (1996). The parameter η is a small constant introduced to regularise singularities that arise when $r_{ab}=0$. The term $f_{ak}$ denotes the interaction force between particle a and wall particle k. It needs to be non‐zero only near boundary walls (refers to Cleary et al. 2021 and Cummins et al. 2012 for further details), and g denotes the gravitational acceleration. Some SPH formulations have reported difficulties in modeling high viscosities. The formulation in Equation (5) does not display such limits with the only restriction being that the explicit time step becomes unpleasantly small at very high viscosities.

Once particle velocities are computed, their position are updated in accordance with Equation (6).

(6) $$\frac{dr_{a}}{dt}=v_{a}+0.5\;\sum_{b}\frac{2m_{b}}{\rho_{a}+\rho_{b}}\left(v_{b}-v_{a}\right)W_{ab}$$

The first term describes the dynamic motion of particles, whilst the second one is a XSPH correction term that contributes to improved numerical stability (Monaghan 1989). The simulations employ an explicit integration scheme, as outlined by Monaghan 1994. It is worth noting that whilst diffusion is explicitly calculated via the first term in Equation (5) convective motion is automatically included via the advection from Equation (6). This means that SPH is a Lagrangian method with these equations solved in the frame of the particles. This means that high speed flows can be handled easily without special treatments for numerical stabilization of advection (as is common in grid and mesh‐based methods with up winding schemes).

The time step is determined using a modified Courant condition that incorporates the effect of viscosity to maintain simulation stability. The specific explanation of this adjustment is detailed in Cleary (1998) and is expressed as:

(7) $$\Delta t=\min_{a}\left(\frac{0.5h}{c_{s}+2\kappa \mu_{a}/h\rho_{a}}\right)\;$$

A particle size of 1 mm is found to be sufficient for capturing the fluid dynamics, yielding a total of approximately 400,000 particles for the fluid domain. Sensitivity of solution accuracy has been investigated with the spatial resolution study can be found in the supplementary material (Figures S1 and S2). This study provides a more refined solution compared to the previous model (Harrison et al. 2018), which used a particle size of 3 mm.

One of the key strengths of SPH for stomach content modeling is that every SPH particle can have different properties including being solid, semi‐solid or liquids of various types. This means that it can be used for mixtures of very different materials and for materials with significant heterogeneity. It is also very well suited to inclusion of reaction chemistry and phase change (Cleary et al. 2021).

#### 2.1.2 Acid Diffusion

The diffusion of gastric acid in the gastric fluid is simulated using a Fick's diffusion model. Instead of simulating actual acid secretion with added mass, this model focuses solely on chemical diffusion. Since the gastric content in this study is purely liquid, simulating only diffusion is reasonable, as the acid won't react with the gastric content. The effect of temperature is not considered in this study, since the range of temperature variation is small. The species transfer equation used in the SPH model is (Harrison et al. 2014):

(8) $$\frac{dC_{a}}{dt}=\sum_{b}\frac{4m_{b}}{\rho_{a}\rho_{b}}\;\frac{D_{ma}D_{mb}}{D_{ma}+D_{mb}}\;\times \;C_{ab}\frac{r_{ab}\;\times \;\nabla_{a}W_{ab}}{r_{ab}^{2}+\eta^{2}}$$

where $C_{a}$ is the concentration of the acid in particle *a*, $C_{ab}$ is the difference in concentration between particles *a* and *b*, $D_{ma}$ and $D_{mb}$ are the mass diffusion coefficients for the materials represented by particles *a* and *b*, respectively. The kinematic diffusion coefficient is given by $D_{a}=D_{ma}\;/\rho_{a}$. The acid concentration is then converted into the pH scale by:

(9) $$\mathrm{pH}=-\log_{10}\left[\frac{C_{a}}{M}\right]$$

where M is the molar mass of hydrogen (1.008 kg/kmol).

#### 2.1.3 Wall Motion

Instead of a stationary mesh, the boundary of the stomach is represented by a sequence of deforming meshes, which represent the temporal variation of the stomach surface position. A constant mesh update period of 0.25 s is used, with deformation between mesh updates given by quadratic interpolation of the position, velocity and normal of each mesh node calculated at each time step. Pylorus dynamics are available in the model, but the pylorus is kept closed for the entire simulation to study the predominant stomach conditions, which is when gastric emptying is not occurring.

### 2.2 Model Configurations

#### 2.2.1 Baseline Configuration

**FIGURE 1:**
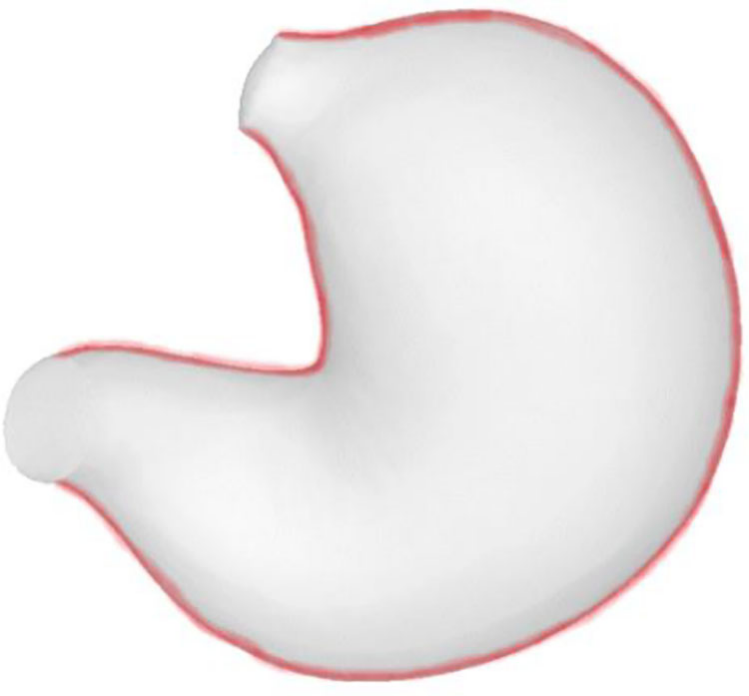
Illustration of the acid diffusion region on the stomach wall, coloured in red.

A generic stomach shape is used in this study, as shown in Figure 1, which has been previously utilized previously by Harrison et al. (2018) and Liu et al. (2023b). The geometry features a total volume of 580 mL, a surface area of 0.04 m^2^, a fundus diameter of 80 mm, and a greater curvature measuring 320 mm, all of which are typical of adult stomachs (Csendes and Burgos 2005; Kim et al. 2020). The model geometry measures, 122 mm in height, 137 mm in width and 122 mm in depth. The peristaltic waves, known as antral contraction waves or ACWs, are modeled as kinematically prescribed deformations of the stomach wall. They emerge at the proximal antrum and approach the pylorus. The parameters defining the ACWs are consistent with those used by Harrison et al. (2018), which were based on the study by Pal et al. (2004). In the base model, the stomach's occlusion is defined to increase from 40% at the fundus to 60% through the antrum and then reach 90% at the pylorus. Each ACW travels down the stomach at a speed of 2.2 mm s^−1^ along the centerline, with an ACW period of 20 s.

The simulation starts with the stomach filled with quiescent fluid at 50% of its capacity, which leads to a total volume of 400 mL. Gravity is applied in the vertical downward direction. The fluid in the stomach has an initial neutral pH of 7. A constant pH boundary condition of 3 is used on the stomach wall, as illustrated in Figure 1. The acid is secreted throughout the mixing process (0 to 300 s) whenever the fluid contacts with the stomach wall, without any additional mass being introduced into the fluid. Both the fluid and the acid are assumed to have a constant density of 1000 kg m^−3^ and a constant dynamic viscosity of 0.1 Pa·s, which is consistent with the representative range of fluid properties found in the stomach. In this study, a diffusion coefficient for H^+^ into water of 10^−9^ m^2^ s^−1^ is used for the base model, based on the literature (Li and Jin 2021; Abrahamsson et al. 2005).

**FIGURE 2:**
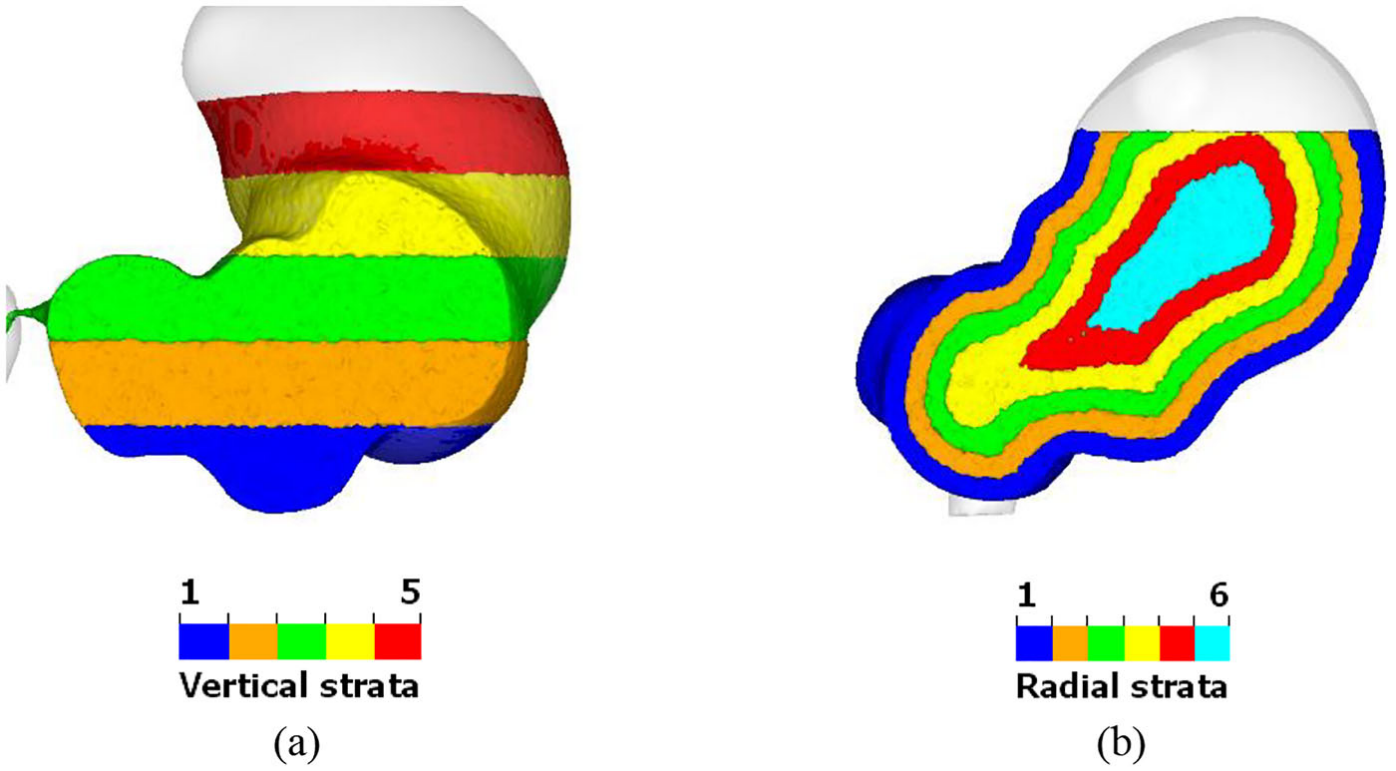
Visualization of initial fluid bands at a cross‐section, with colors representing initial positions; (a) vertical (blue = bottom layer, green = middle and red = top); and (b) radial (blue = outermost, green = middle and cyan = innermost).

As in our previous work (Liu et al. 2023b), the initial fluid volume is again divided into five bands with the same height, and different colours are ascribed to the particles in each layer, as shown in Figure 2. One set of bands is separated vertically (Figure 2a), while the other is separated “radially” (Figure 2b) according to the normal distance from the stomach wall. The fluid particles retain their initial colour when the fluid is mixed, allowing the mixing behaviour to be visualised and analysed. The average height of each set of coloured particles is calculated and recorded throughout the simulation.

The state of mixing at each time point, ξ, is characterised by calculating the mean range of particle colours in each 1 cm^3^ cell of a cubic grid that encompasses the volume of the stomach.

(10) $$\xi =1-\frac{\sigma \;-\;\sigma_{\min}}{\sigma_{\max}\;-\;\sigma_{\min}}$$

where σ is the grid‐averaged value of the standard deviation of particle colour over all of the cells. The terms $\sigma_{\max}$ and $\sigma_{\min}$ represent the maxium and minimum possible values of σ for a given SPH particle resoltion and are determined from numerical simulation. This means that 0<ξ<1 with ξ=1 being fully unmixed and ξ=0 being perfectly mixed.

The mixing performance is then evaluated by plotting the state of mixing ξ against time. A modified exponential curve, given in Equation (6) below, is used to fit the mixing data, similar to its use in describing gastric emptying data (Elashoff et al. 1982). Gastric half‐emptying time is widely used to evaluate the efficiency of gastric emptying between different meals. The same concept is applied here in quantifying the efficiency of mixing with different gastric motility conditions, via

(11) $$f_{mix}=1-2^{-\frac{t}{t_{1/2}}}$$

where $t_{1/2}$ is the half‐mixing time when the gastric content is 50% mixed.

#### 2.2.2 Parameter Variations

The sensitivity of the model to input parameters is studied, as listed in Table 1, 2, to understand how their variations affect gastric mixing and acid diffusion. The parameters tested include fluid viscosity, occlusion and speed of the ACW, and acid diffusion rate, all of which are all closely related to food digestion in the stomach. Dynamic viscosities of 0.02, 0.1 and 0.5 Pa·s are examined, representing the viscosities of starch syrup (Kozu et al. 2010), apple juice (Garcia et al. 2005), and honey at 45°C (Yanniotis, 2006), respectively. The motility index (MI) is a quantitative measure used to evaluate antral motility. It is calculated by multiplying the mean occlusion by the frequency of contractions (Sogabe et al. 2005). Mean occlusion is defined as the ratio of the difference between the relaxed (original) volume of the stomach without ACWs and the contracted volume with ACWs to the relaxed volume. An increase in MI indicates enhanced gastric motility, meaning the stomach's ability to contract and move food through the digestive tract is improved. Conversely, a decrease in MI is often associated with gastroparesis, a condition where the stomach's motility is impaired (Kloetzer et al. 2010).

**TABLE 1:** Parameter variations for the model sensitivity analysis.

Name	Viscosity (Pa·s)	Relative antral contraction wave (ACW) speed	Relative occlusion	Relative motility index (MI)	Acid diffusion rate (m^2^ s^−1^)
Baseline	0.10	1.0	1.0	1.0	1 × 10^−9^
0.02 Pa·s	0.02	1.0	1.0	1.0	1 × 10^−9^
0.5 Pa·s	0.5	1.0	1.0	1.0	1 × 10^−9^
Slower	0.1	3/5	1.0	0.6	1 × 10^−9^
Faster	0.1	5/3	1.0	1.67	1 × 10^−9^
75%	0.1	1.0	0.75	0.75	1 × 10^−9^
125%	0.1	1.0	1.25	1.25	1 × 10^−9^
Low	0.1	1.0	1.0	1.0	1 × 10^−10^
High	0.1	1.0	1.0	1.0	1 × 10^−8^

Viscosity values correspond to starch syrup (Kozu et al., 2010), apple juice (Garcia et al., 2005), and honey at 45° C (Yanniotis, 2006).

By varying the occlusion and the speed of the ACWs, we can investigate the relationship between gastric motility and mixing. MRI and electrophysiology experiments have shown variations in wave frequency and amplitude (O'Grady et al. 2010; Kwiatek et al. 2006; Berry et al. 2016). To investigate the effect of gastric motility on mixing performance, simulations are performed in which the ACW speed is varied by symmetric scaling factors of 3/5 and 5/3 of the baseline case. Additional simulations are performed in which the overall occlusion is modified by increasing or decreasing it by 25% across all regions of the stomach. The resulting relative MIs range from 0.6 to 1.67.

Diffusion coefficients in liquids vary significantly based on temperature, viscosity, and composition (Cussler 2009). Higher temperatures and lower viscosity facilitate faster diffusion, while more viscous or complex liquids, such as juices, slow it down. To study the diffusion of gastric acid into different liquids, the diffusion coefficient is varied from 10^−10^ to 10^−8^ m^2^ s^−1^, accounting for these factors. Within each case, the diffusion coefficient is assumed to be constant, indicating that acid molecules spread uniformly regardless of the fluid's viscosity. This simplification was made to enable separation of the basic transport dependence of acid on diffusivity. The effect of viscosity dependence of the diffusivity on the transport will be considered in future work. Each simulation was run for 5 min of simulation time with the baseline model taking approximately 10 days to complete using 36 cores of Intel Xeon Gold 6154 CPUs operating at a clock speed of 3.0 GHz.

## 3 Mixing and Acid Transport

### 3.1 Fluid Mixing Behaviour

**FIGURE 3:**
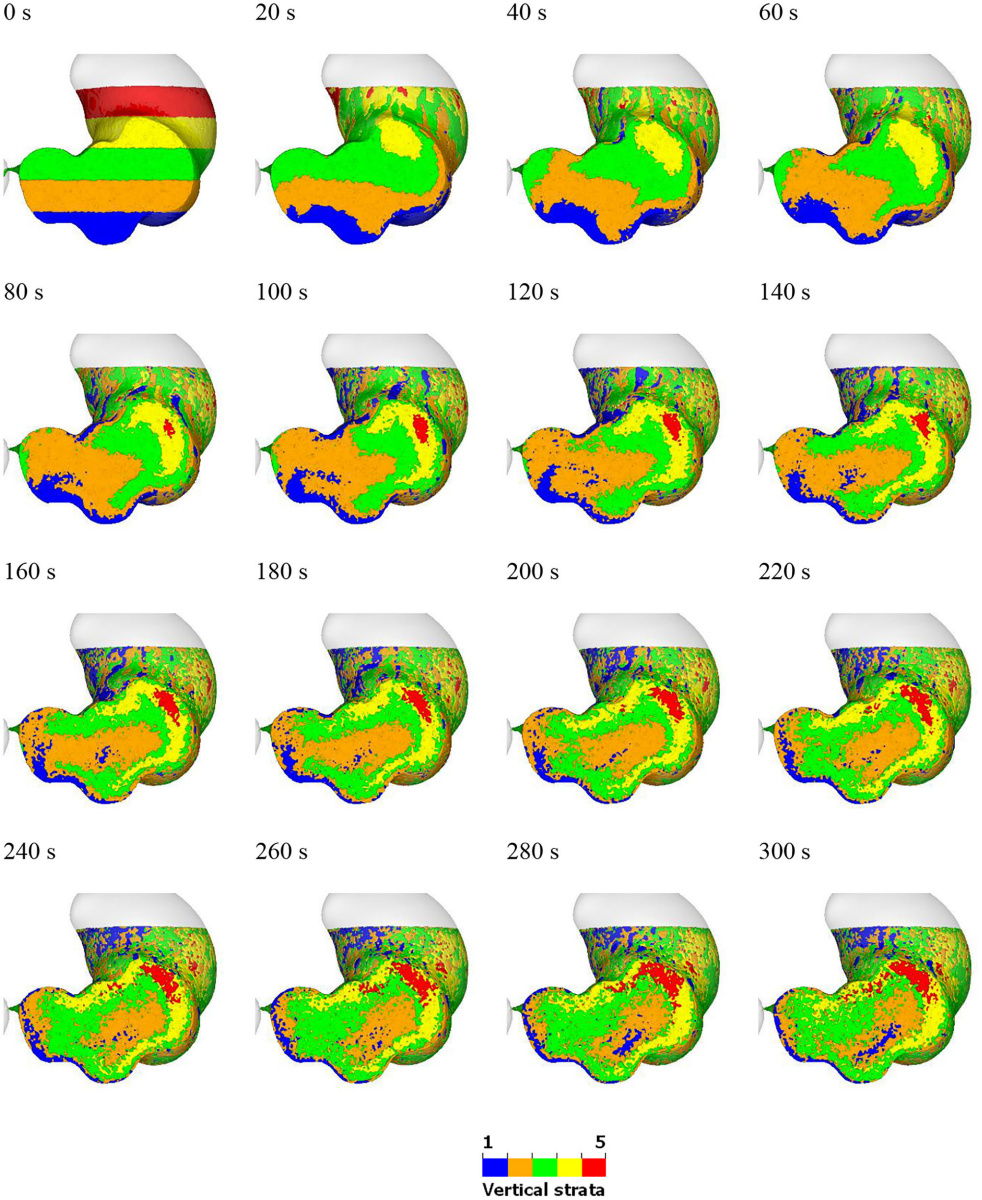
Visualization of the mixing behavior within the stomach. Fluid particles are coloured by their initial vertical positions (blue = bottom layer, green = middle layer and red = top layer).

**FIGURE 4:**
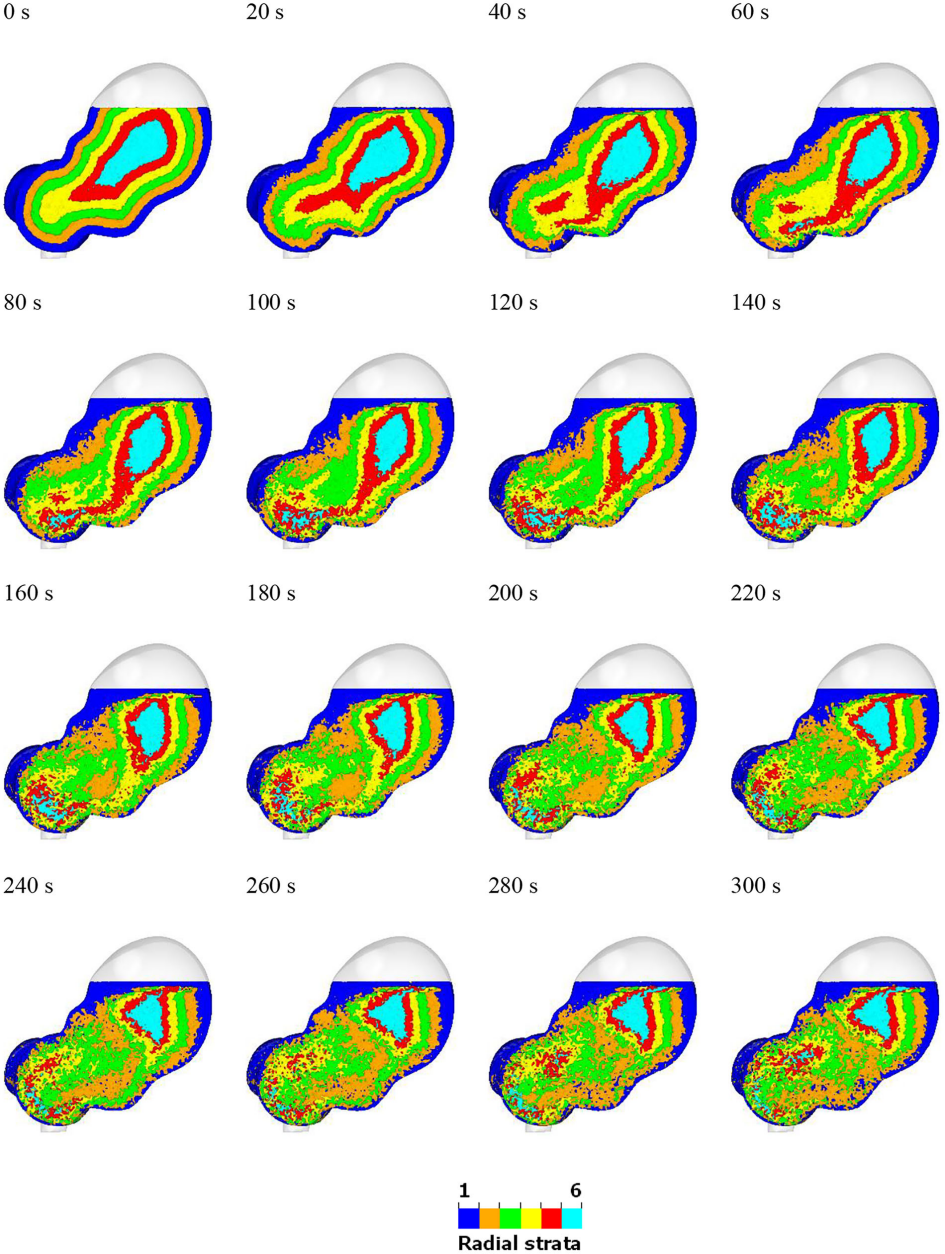
Visualization of the radial mixing behavior within the stomach. Fluid particles are coloured by their initial radial positions (blue = outermost layer, green = middle layer and cyan = innermost layer).

The mixing behavior of the fluid in the stomach is illustrated in Figures 3 and 4. Figur  3 shows the vertically stratified bands at the start of the simulation. As the ACWs propagate toward the pylorus, the blue‐coloured fluid at the bottom (layer 1) is gradually pushed along the stomach wall. By 40 s, some of the blue fluid has reached the free surface. At 80 s, blue fluid starts to appear at various locations along the stomach wall. From 100 s onward, some of it moves toward the center of the stomach, although most of it remains near the upper and lower walls even up to 300 s. The orange fluid (layer 2) predominantly stays in the lower region of the stomach from 0 to 140 s. Between 160 and 300 s, while parts of the it spread to different locations along the wall, the majority appears to be trapped in the center of the stomach. Similarly, the green fluid (layer 3) remains in the upper region from 0 to 60 s. From 80 to 300 s, it moves around the orange fluid, eventually enveloping it. This suggests efficient transport parallel to the stomach walls but poor transport away from them. The yellow fluid (layer 4) moves along with the ACWs, gradually flowing along the greater curvature (lower right of the stomach wall) from 0 to 140 s. From 140 to 300 s, it also moves along the lesser curvature (upper left of the stomach wall). The red fluid (layer 5) begins to appear at the cross‐section at 80 s, trapped within the yellow fluid and near the origin of the greater curvature. It gradually accumulates in this location from 80 to 180 s and then slowly spreads along with the yellow fluid. Overall, the fluid dynamics within the stomach, as driven by the ACWs, show a complex pattern of movement and mixing. After 200 s, there is a good level of macro‐mixing of all layers of fluid.

**FIGURE 5:**
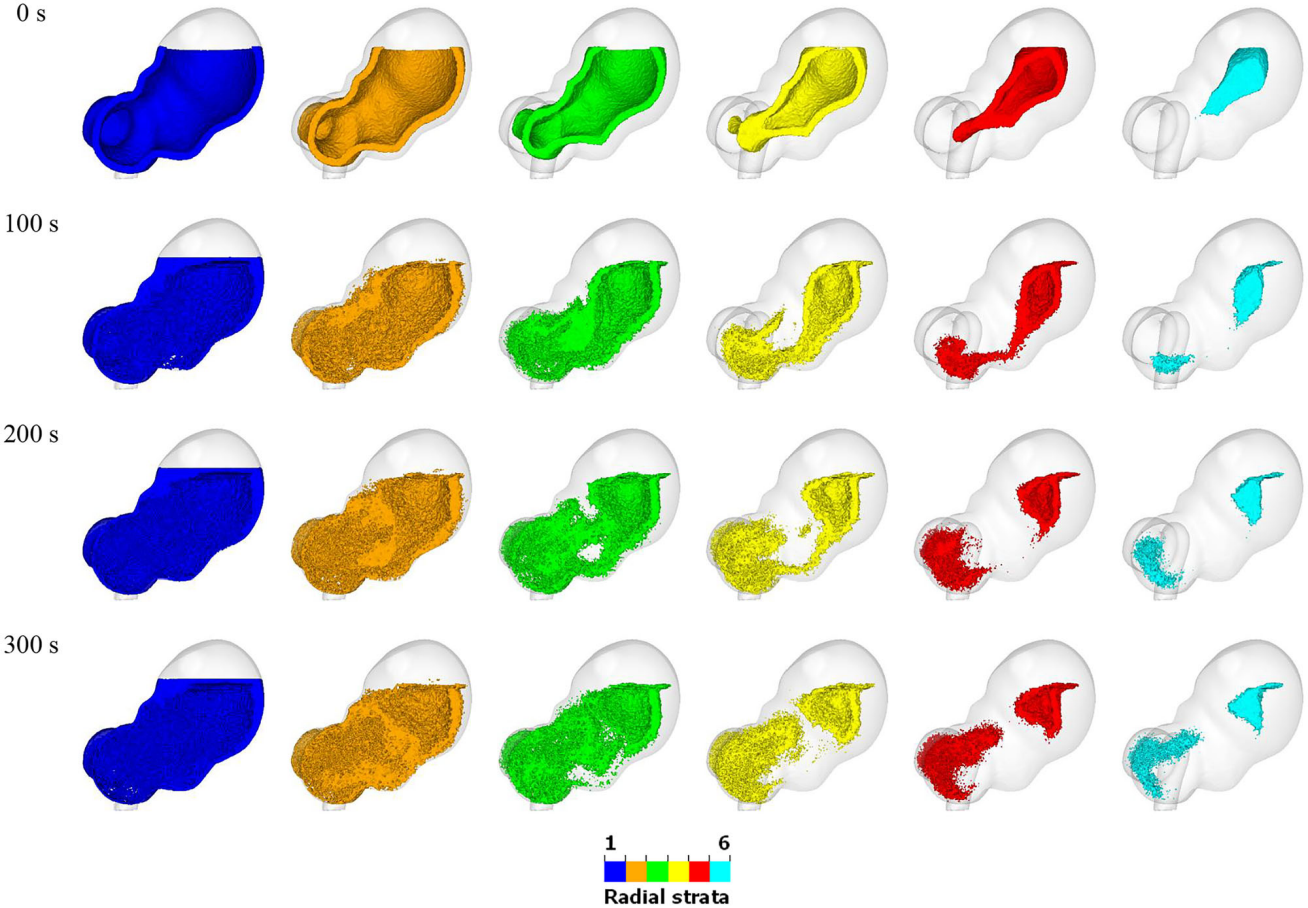
Visualization of the transport of each separate radial band during 300 s of simulated mixing.

Radial strata for the same simulation are shown in Figure 4 (all color bands) and Figure 5 (individual color band). There is minimal radial mixing in the upper region of the stomach, while significant mixing occurs in the lower region due to the ACW. This observation corresponds with the ACW's motion, where the occlusion starts at the fundus (40%), increases to 60% at the antrum, and reaches 90% at the pylorus. The blue fluid remains near the stomach wall throughout the mixing process, gradually accumulating at the free surface from 40 s onward and enveloping the other fluids over time. The position of the orange fluid remains relatively unchanged. The inner fluids, including the green, yellow, red, and blue layers, separate into two distinct parts after 100 s. This observation is obvious in Figure 5. The mixing in the upper region remains stable, showing little variation over time. However, the mixing in the lower region is more robust, with significant changes in the mixing pattern. This observation matches well with the classical description of gastric function. The proximal stomach, which includes the fundus and corpus, serves as a reservoir where undigested food is stored (Urbain et al. 1989). The distal stomach, consisting of the antrum and pylorus, serves as a grinder to break down large food particles. It also acts as a pump by generating peristaltic waves in the stomach wall, which promote gastric motility and facilitate the movement of gastric fluids within the stomach (Brandstaeter et al. 2019; Beckett et al. 2003).

The large differences between vertical and radial mixing behaviors highlight that mixing is predominantly achieved through transport parallel to the walls and driven by the tangential motion of the ACW structures. While vertical bands are affected by tangential transport, radial bands are not. Consequently, there is significant mixing in the form of shear deformation of layers parallel to the wall, which the ACW waves effectively facilitate, but this contributes little to radial transport.

### 3.2 pH Changes in the Fluid

**FIGURE 6:**
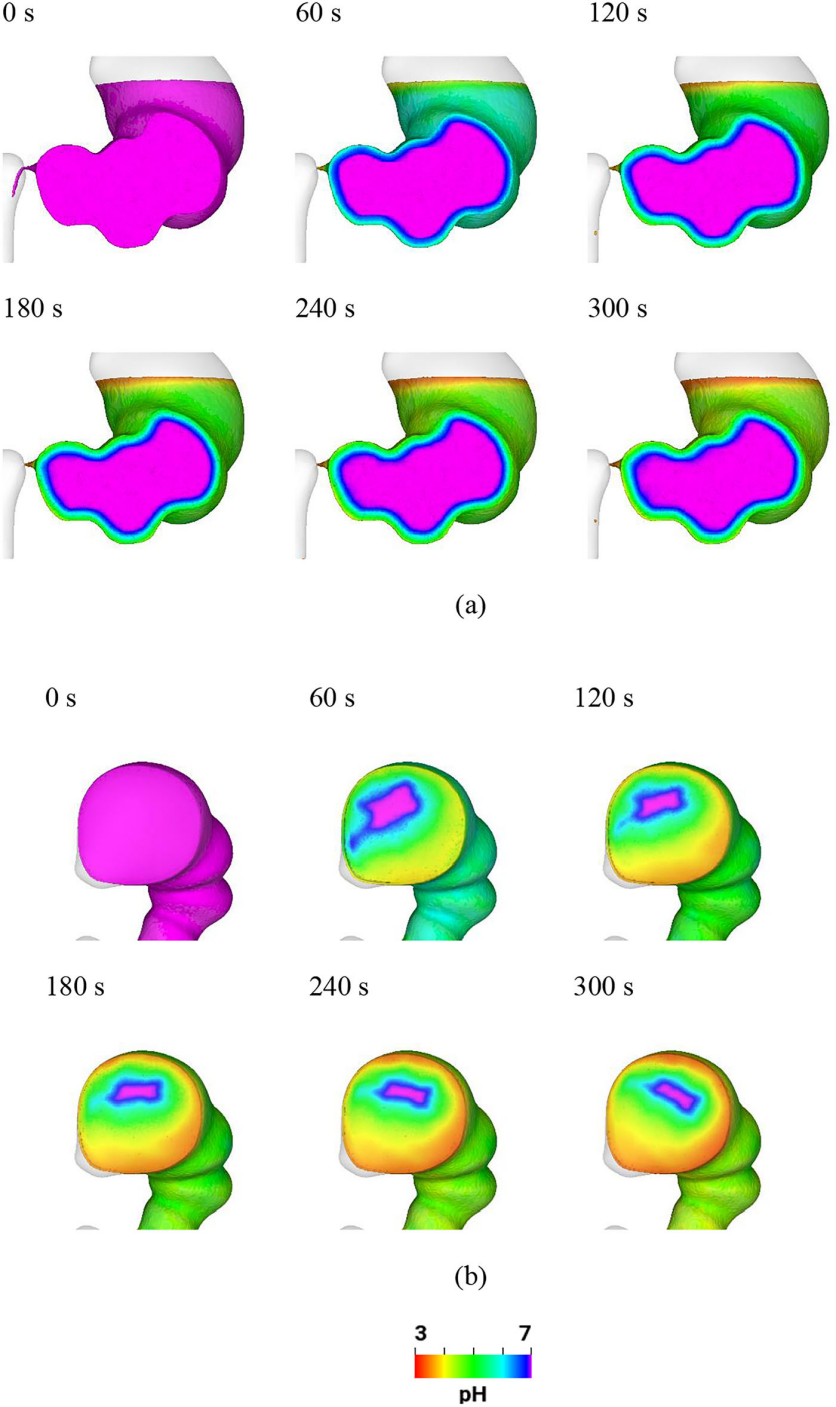
Visualization of the pH changes in the fluid from 0 s to 300 s (a) front view; and (b) top view (purple: pH = 7, green: pH = 5; red: pH = 3).

The change in pH of the fluid in the stomach over time is shown in Figure 6. In this figure and all figures showing pH changes in the fluid, the boundary particles (prescribed with a constant pH of 3) are not shown. A purple color band with a pH of between 6.8 and 7.0 is used to identify the front of the acid ingress. The thickness of the acid‐rich layer, defined as the region between the inner boundary of the purple fluid volume and the stomach wall where the pH is below 6.8, is recorded for further analysis. This thickness is averaged over the wetted surface of the stomach lining and represents the indicative distance at which the acid concentration reaches a pH of 6.8. Initially (at 0 s) the pH of the fluid is 7.0 (neutral). Throughout the mixing process (0 to 300 s), acid is secreted from the stomach wall and penetrates in a primarily radial direction into the gastric fluids, suggesting that acid ingress is primarily driven by diffusion alone. Thus, the ACW movements may have limited effects on acid distribution in most of the stomach, despite the radial mixing in the lower stomach shown in Figure 4. The strong free surface mixing shown in Figure 4 results in significant advection of acid into the top layer. Similar findings were observed in vivo by MRI (Steingoetter et al. 2015), single‐photon emission computed tomography (Kuiken et al. 2002), and in vitro (Fletcher et al. 2001) methods, specifically showed that the gastric secretion forms a layer in the proximal stomach after ingestion of a liquid meal (Steingoetter et al. 2015). The accumulation of acid at the top layer highlights the dynamic nature of acid distribution within the stomach during mixing, particularly in the proximal stomach where advection plays a more prominent role compared with diffusion. With a low pH approaching 3, this acid activates digestive enzymes like pepsinogens, essential for protein digestion (Yamada et al. 2003). As the acid at the top is close to the esophagogastric junction, it can flow back into the esophagus if the lower esophageal sphincter is weak or relaxes inappropriately, causing gastro esophageal reflux symptoms (Steingoetter et al. 2015; Kuiken et al. 2002).

**FIGURE 7:**
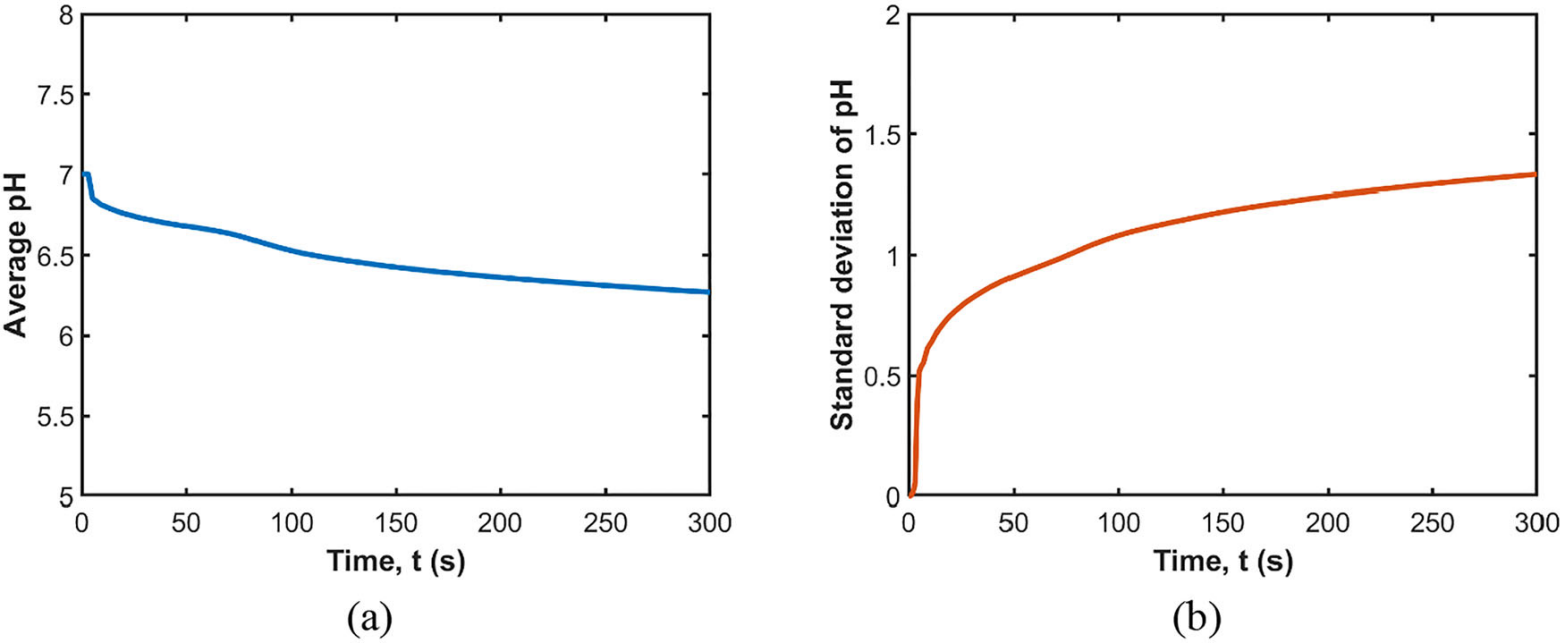
(a) Average and (b) standard deviation of pH in the stomach over time.

Figure 7 shows the average and standard deviation of pH in the stomach fluid during 300 s of mixing. Initially, the average pH is neutral at 7.0. With the acid secreted constantly at a pH of 3, the average pH gradually decreases to 6.3 over 300 s, indicating increased acidity. The standard deviation starts from 0 and reaches 1.3 at 300 s. This variability, along with the accumulation of acid at the free surface and its penetration into the internal fluid, highlights the dynamic and strongly spatially inhomogeneous nature of the acid distribution within the stomach during mixing. The increased standard deviation suggests that different regions of the stomach experience varying levels of acidity, which influences the activation of digestive enzymes and the breakdown of food particles in a non‐uniform manner.

## 4 Model sensitivity analysis

In this section, we delve into the detailed impact of various input parameters on gastric mixing and acid dissolution. Specifically, we examine how fluid viscosity, ACW speed, relative occlusion, and acid diffusion coefficient influence these processes.

### 4.1 Effect of fluid viscosity

**FIGURE 8:**
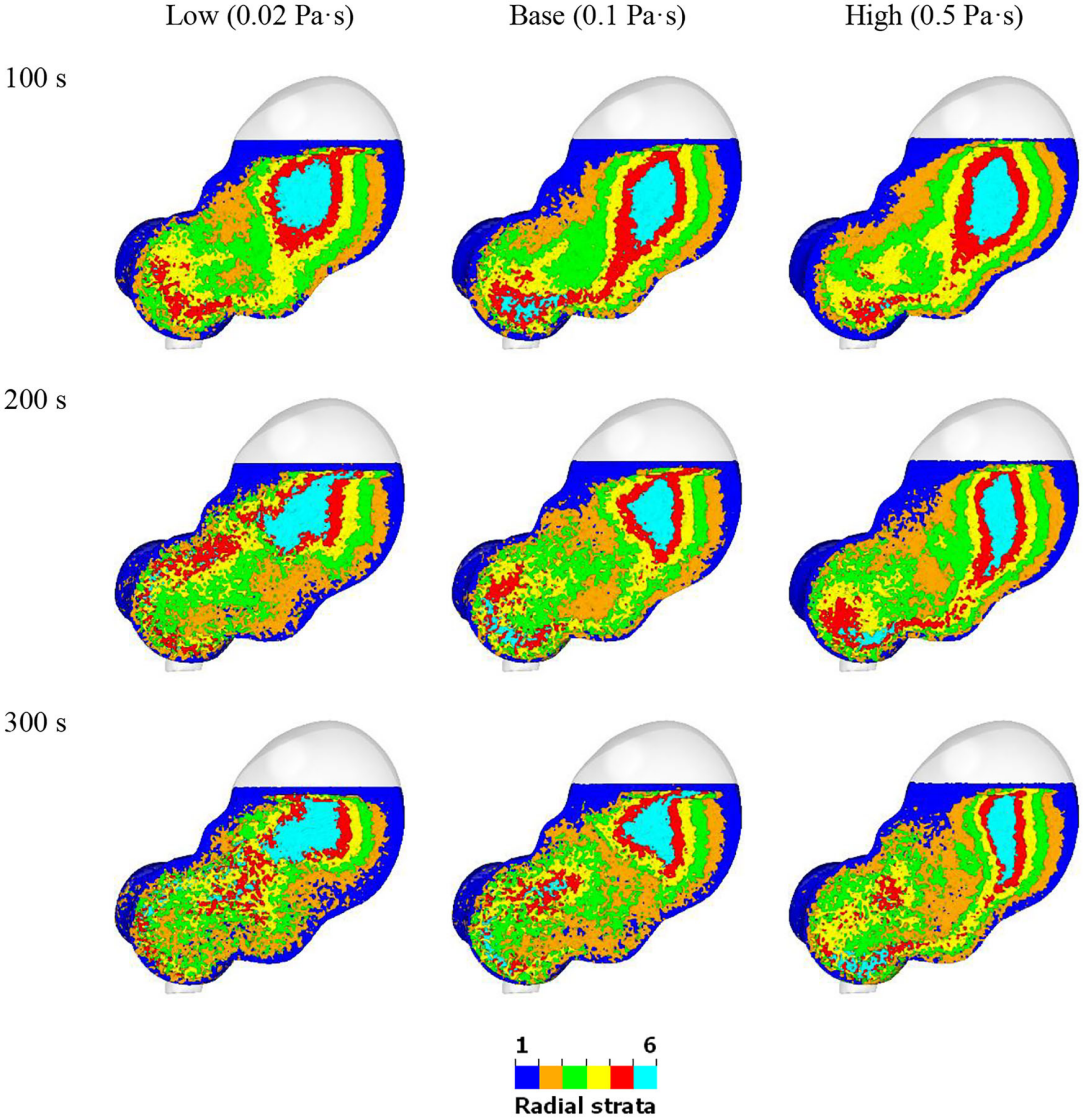
Visualization of radial mixing for different fluid viscosities at 100 s, 200 s, and 300 s. (left) 0.02 Pa·s; (middle) 0.1 Pa·s; (right) 0.5 Pa·s.

The mixing behavior of fluids with different viscosities at 100, 200, and 300 s is shown using radial strata in Figure 8. For lower viscosity fluids, the mixing is more constrained to the lower region of the stomach, with the upper region's strata appearing relatively undisturbed. As viscosity increases, more of the upper stomach is pulled into the mixing flow, but the overall level of mixing decreases. At 300 s, it is evident that the 0.5 Pa·s fluid near the greater curvature (the long convex lateral border of the stomach) follows the same trajectory as the ACW, while this is less apparent for the low‐viscosity fluids. The mixing rate for the interior fluid is much faster with the low‐viscosity fluids. At 300 s, the interior of the 0.02 Pa·s fluid, located in the middle of the stomach, shows a good mix of different color bands. Compared with the fluid in the proximal stomach, the mixing in the distal stomach is much more efficient for fluid with all viscosities.

**FIGURE 9:**
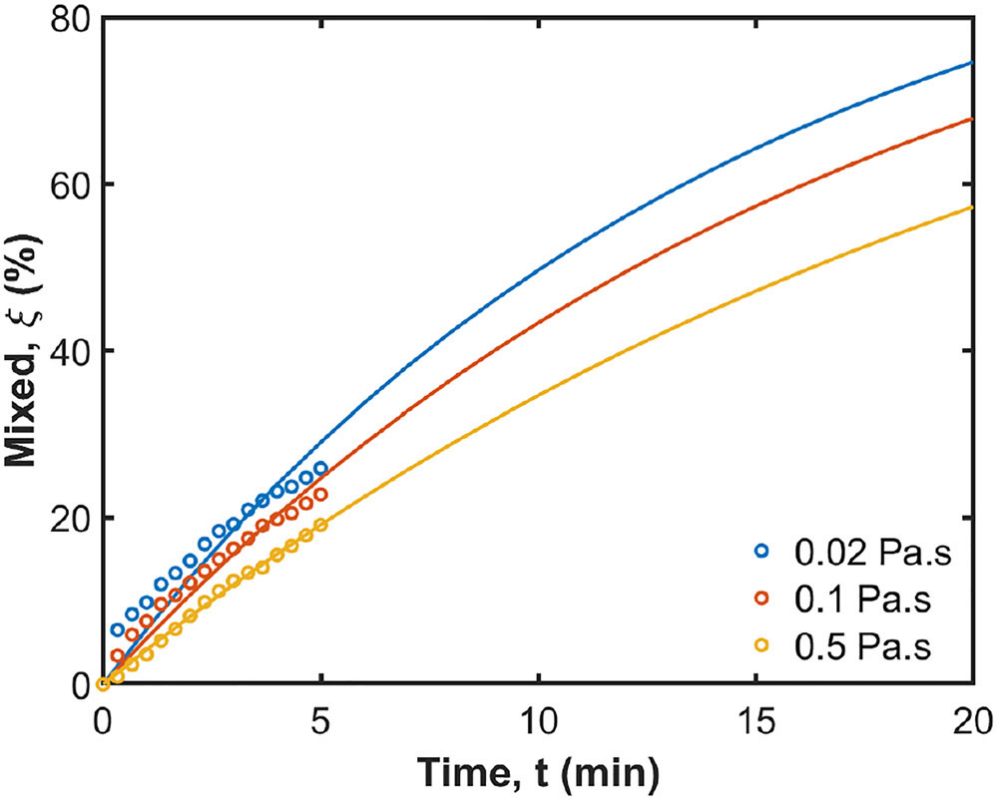
Quantification of radial mixing for different fluid viscosities. Simulation results are shown with circles from 0 min to 5 min, and exponential extrapolations are shown as solid lines from 0 min to 20 min.

Figure 9 quantifies radial mixing for fluids with different viscosities by presenting $t_{1/2}$ and the coefficient of determination ($R^{2}$) for each viscosity. Each curve follows an exponential trend, with the mixing percentage starting at 0% and reaching 26%, 23%, and 19% at 5 min (300 s) for 0.02 Pa·s, 0.1 Pa·s, and 0.5 Pa·s, respectively. They are predicted to reach 75%, 68%, and 57% at 20 min. This indicates that lower viscosity fluids mix more rapidly compared to higher viscosity fluids (which is consistent with what was observed in Figure 8). The rapid mixing of the 0.02 Pa·s fluid suggests that it is more easily influenced by the ACW movements, leading to a more homogeneous distribution within the stomach. In contrast, the higher viscosity fluids, exhibit slower mixing rates, resulting in less uniform distribution. Similar findings were found in the computational studies conducted by Kozu et al. (2010) and Alokaily et al. (2019). This difference in mixing behavior highlights the significant impact of fluid viscosity on the efficiency of the mixing process within the stomach.

To quantify the efficiency of mixing, $t_{1/2}$ of fluids with different viscosities is compared in Table 2. The $R^{2}$ value of each curve is close to 1.00, suggesting a strong correlation between the model predictions and the observed data. $t_{1/2}$ for radial mixing is 10.2 min for 0.02 Pa·s, 12.2 min for 0.1 Pa·s, and 16.3 min for 0.5 Pa·s. While the mixing time of pure liquids is not available in the literature, we use the half‐emptying times as a comparative measure to understand the timescale of gastric mixing. This approach is justified because the ACWs control both gastric mixing, and the emptying processes (Miyagawa et al. 2016; Ebara et al. 2023) and the emptying phase starts after the gastric mixing (propulsion) phase is completed (Bellmann et al. 2016). Most gastric emptying data from the literature are for complete meals containing solid foods, while data for pure liquids are relatively limited. As a reference, the half‐emptying time for water is approximately 10 min (Goyal et al. 2019). For a liquid with a viscosity of 0.02 Pa·s, the half‐emptying time is around 17 min (Marciani et al. 2000), and for a liquid with a viscosity of 1.1 Pa·s, it is about 19 min (Marciani et al. 2000). In an in vivo human study, the half‐emptying times for orange juice ranged from 14 to 19 min depending on its temperature. These results indicate that the mixing timescales in our model are consistent with the gastric emptying times reported in the literature, with lower viscosity fluids mixing and emptying occurs more rapidly than mixing for higher viscosity fluids. However, it is important to acknowledge certain limitations of the current model. Specifically, the absence of pyloric discharge mechanisms and the lack of food breakdown in these simulations affect the generality of the conclusions on gastric mixing dynamics.

**TABLE 2:** Comparison on half‐mixing time with different fluid viscosities.

Variation	Viscosity (Pa·s)	Half‐mixing time $t_{1/2}$ (min)	$R^{2}$
0.02 Pa·s	0.02	10.2	0.89
Baseline	0.1	12.2	0.95
0.5 Pa·s	0.5	16.6	1.00

Viscosity plays a significant role in the mixing dynamics within the stomach. Although the acid diffusion coefficient is expected to vary with viscosity (Cussler 2009), this study assumes it is constant. Figures S3 and S4 indicate that, regardless of fluid viscosity, the thickness of the acid‐rich layer consistently increases from 0.6 mm to 2.2 mm while the pH decreases from 7 to 6.3. Viscosity has a limited effect on pH transport due to the poor radial advection, making diffusion the dominant mechanism for radial chemical transport. The difference between vertical and radial mixing shows that ACWs effectively facilitate vertical mixing but not radial mixing, resulting in low rates of radial transport. Most of the mixing occurs tangentially to the walls and orthogonal to the radial strata in this anisotropic mixing system. The anisotropic nature of mixing can lead to heterogeneous distribution, delayed dissolution, and non‐uniform emptying, all of which negatively impact nutrient absorption and oral drug delivery. Understanding and modeling these patterns is essential for improving formulation strategies and predicting bioavailability in pharmacokinetics and nutrition science.

### 4.2 Effect of Gastric Motility

**FIGURE 10:**
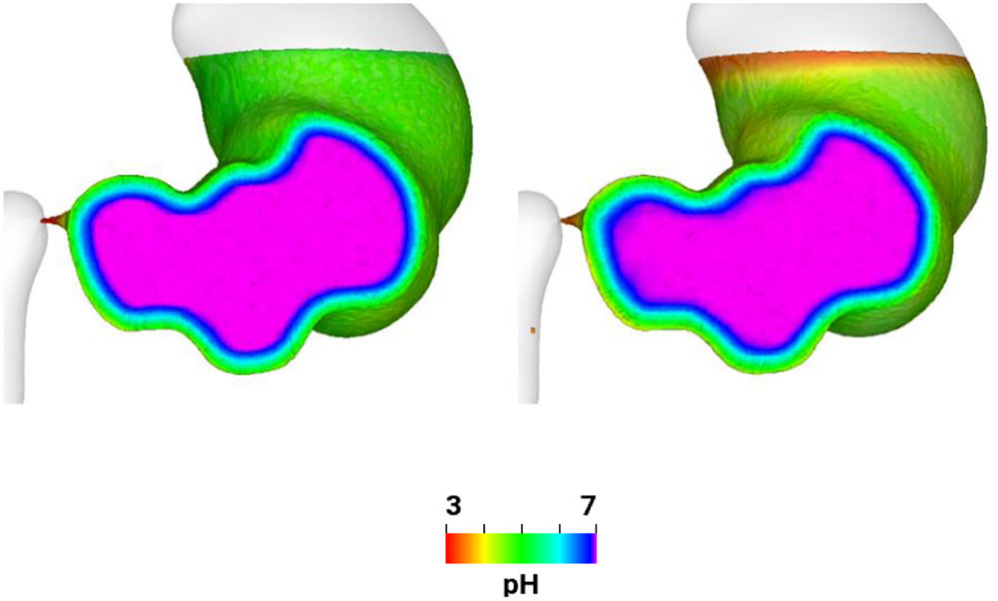
Visualization of pH changes in the fluid for static (left) and moving wall (right) at 300 s.

**FIGURE 11:**
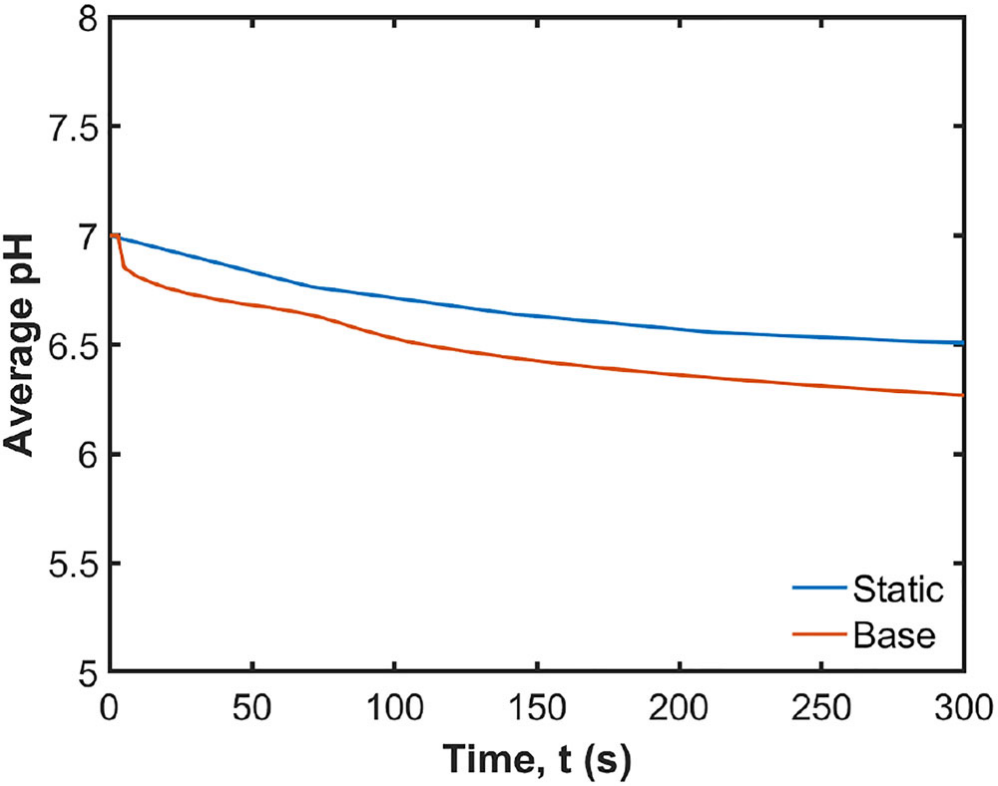
Effect of the moving wall on average pH in the stomach.

**FIGURE 12:**
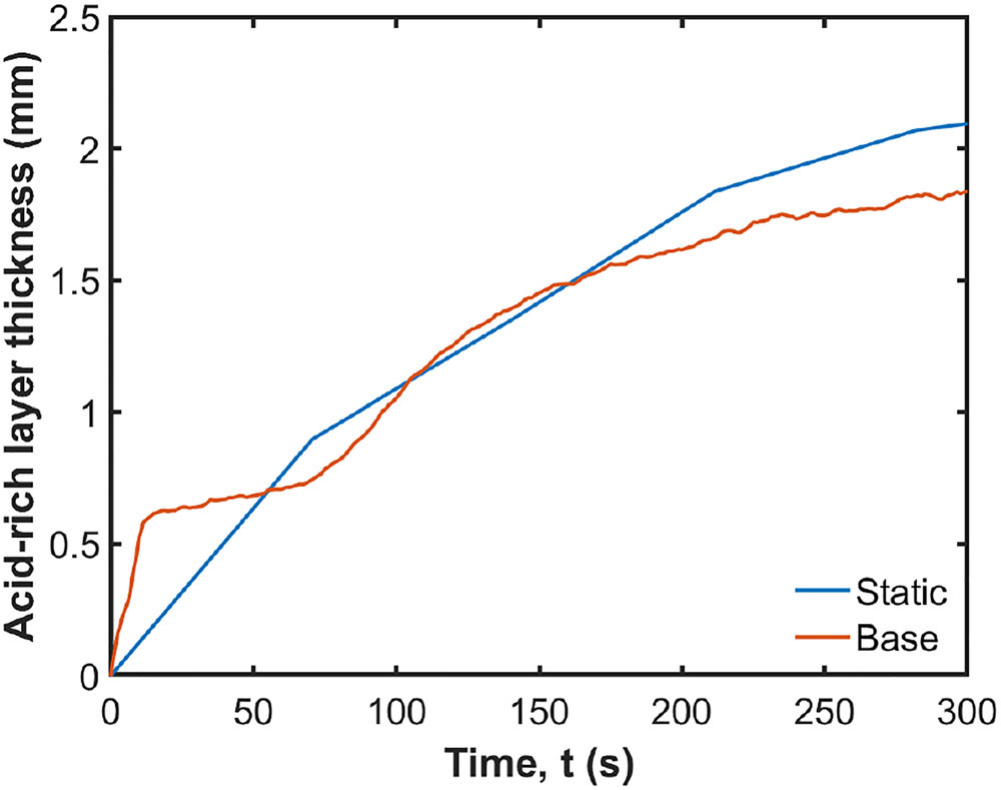
Effect of wall motion on acid‐rich layer thickness.

The effect of the moving stomach wall on the average pH is analyzed by comparing the base case results with a simulation using a static stomach wall. The visualization of the pH changes is shown in Figure 10, and the effect on the average pH is shown in Figure 11. Without wall motion, the acid mass transfer occurs purely by diffusion, reducing the pH from 7.0 to 6.5. With wall motion, the pH decreases further to 6.3. The primary difference is the acid accumulation at the free surface when the ACWs are included (as seen in Figure 10). The ACWs cause the outermost radial layer (blue strata in Figure 4) to move up to the free surface due to tangential advection, resulting in acid accumulation at the free surface. On the other hand, the difference in pH changes is not obvious in the lower region of the stomach, apart from a slight increase in the thickness of the blue and green layers with the moving wall. The advection is dominantly tangential, leading to only very weak radial transport from the walls into this lower region. This is confirmed by Figure 12, which compares the thickness of the acid‐rich layer with pH below 6.8 with and without the wall motion, for which the differences are not significant.

**FIGURE 13:**
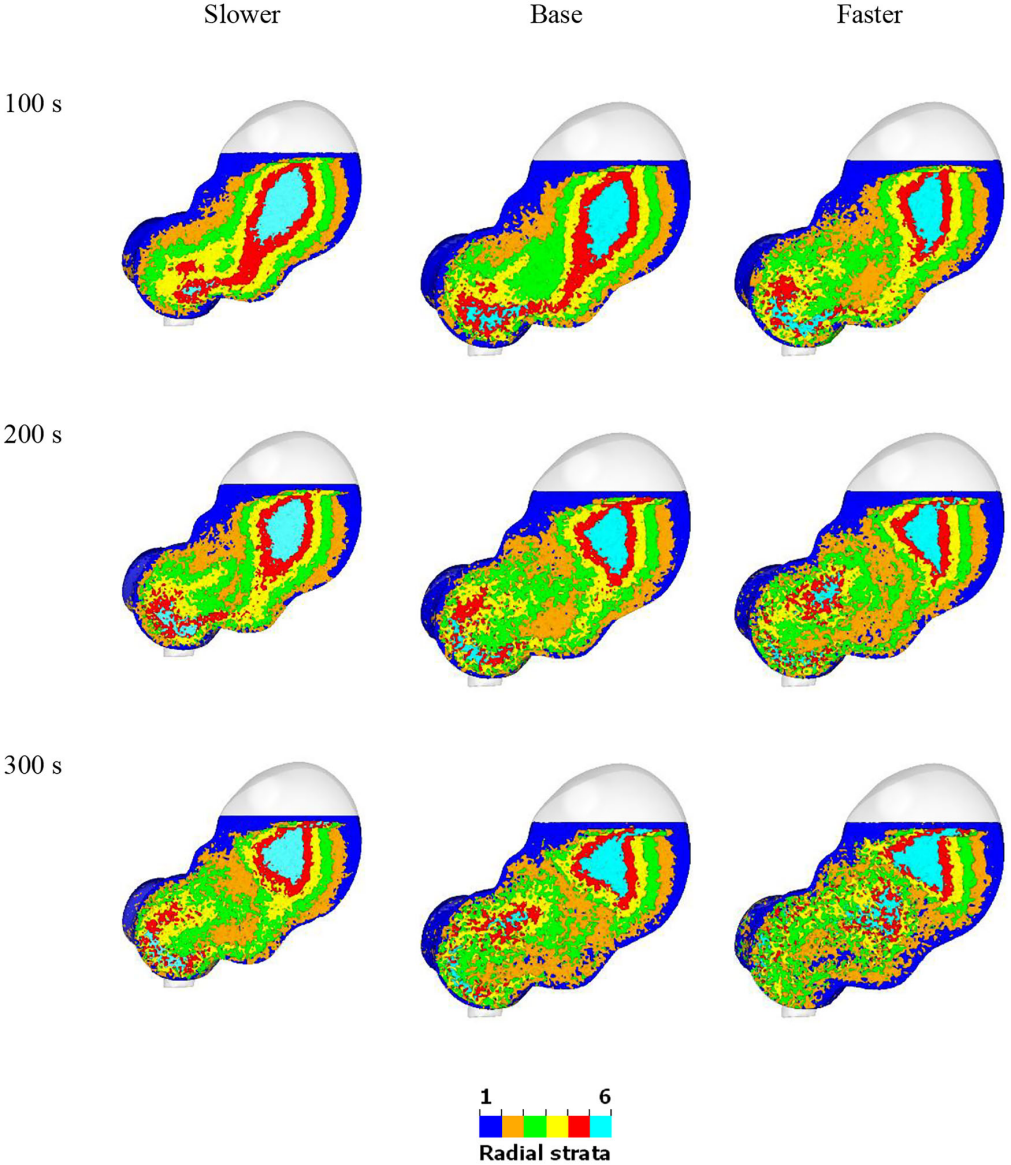
Visualization of radial mixing for different contraction wave speeds at 100 s, 200 s, and 300 s. (left) slower wave; (middle) base; (right) faster wave.

**FIGURE 14:**
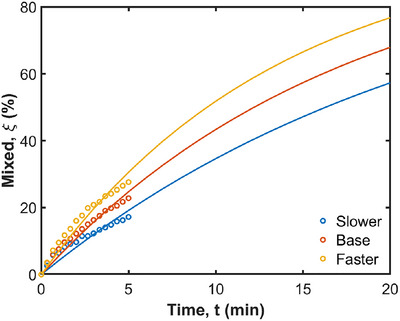
Quantification of radial mixing for different ACW speeds. Simulation results are shown with circles from 0 min to 5 min, and exponential extrapolations are shown as solid lines from 0 min to 20 min.

The speed of the contraction wave is examined by increasing and decreasing the wave speed by factors of 5/3 and 3/5. The mixing behavior of the radial color bands for different contractive wave speeds is shown in Figure 13. With an increased wave speed, the fluid near the ACW moves significantly faster. Initially, it travels along the stomach wall, passing towards the pylorus region. Subsequently, a retropulsive motion is observed, where the fluid near the pylorus moves into the central region. The increased wave speed enhances the mixing behavior, leading to more efficient mixing and distribution of fluid in the lower two‐thirds of the stomach. Additionally, there is a modest increase in the proportion of stomach contents that are well‐mixed as the speed increases. Figure 14 quantifies radial mixing for different ACW speeds. The curves start from 0%, reach 17%, 23%, and 28% at 5 min for faster, base, and slower speeds, respectively, and are predicted to reach 77%, 68%, and 57% at 20 min. As shown in Table 3, $t_{1/2}$ for the slower ACW, base ACW, and faster ACW are 16.3, 12.2, and 9.5 min, respectively. These results indicate that increasing the ACW speed leads to faster mixing, as evidenced by the shorter $t_{1/2}$. Again, the $\;R^{2}$ values suggest a strong correlation between the model predictions and the observed data, indicating reliable quantification of the mixing process across different ACW speeds.

**TABLE 3:** Comparison of half‐mixing time across different gastric motility conditions.

Name	Relative antral contraction wave (ACW) speed	Relative occlusion	Relative motility index (MI)	Half‐mixing time $t_{1/2}$ (min)	$R^{2}$
Baseline	1.0	1.0	1.0	12.2	0.95
Slower	3/5	1.0	0.6	16.3	0.88
Faster	5/3	1.0	1.67	9.5	0.94
75%	1.0	0.75	0.75	15.8	0.97
125%	1.0	1.25	1.25	8.9	0.96

**FIGURE 15:**
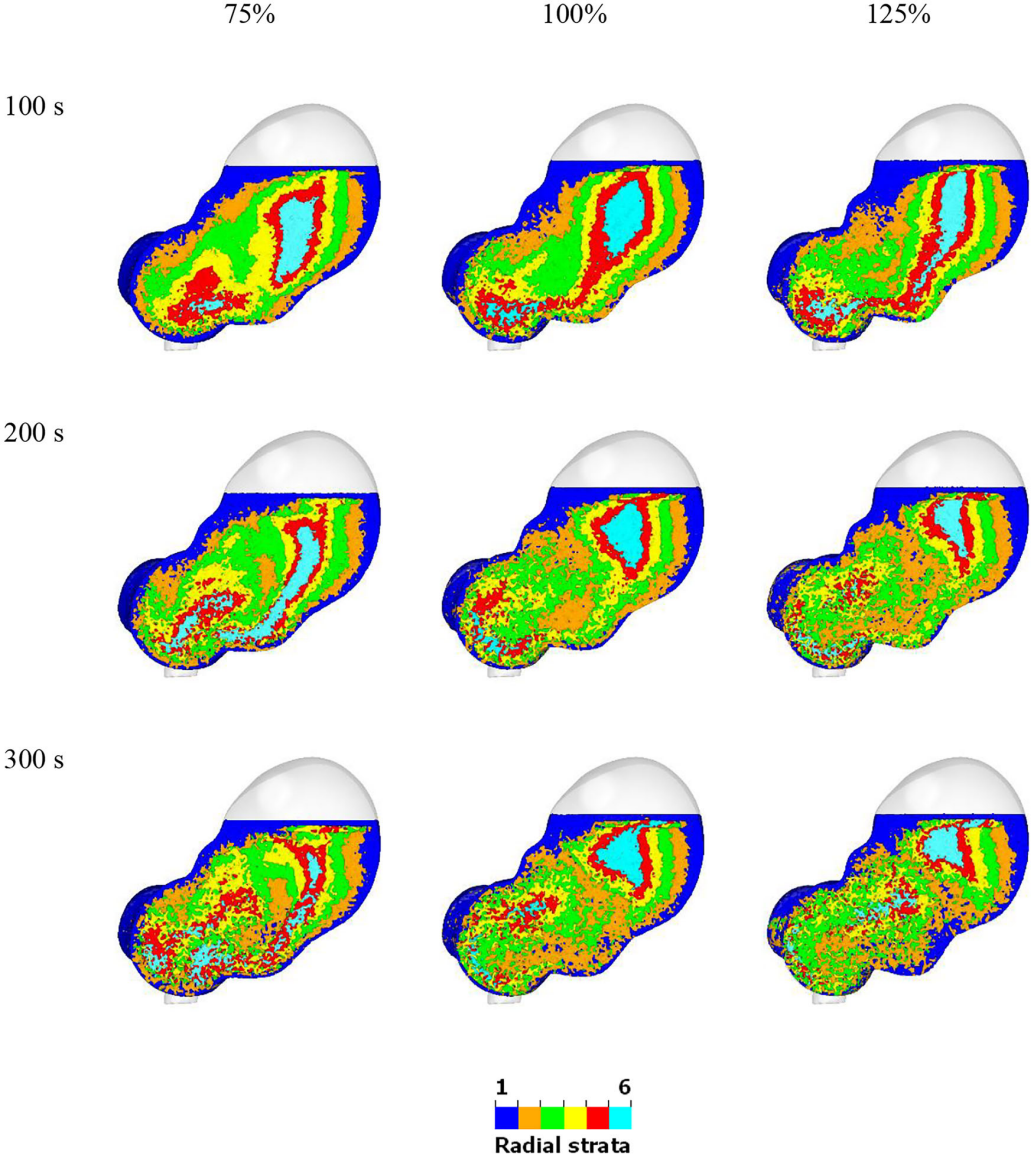
Visualization of radial mixing within the stomach for different relative occlusions at 100 s, 200 s, and 300 s. (left) 75%; (middle) 100%; (right) 125%.

**FIGURE 16:**
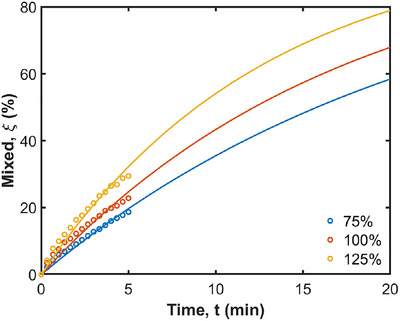
Quantification of radial mixing for different ACW relative occlusions. Simulation results are shown with circles from 0 min to 5 min, and exponential extrapolations are shown as solid lines from 0 min to 20 min.

The effect of changes to relative occlusion of the ACW on mixing and acid diffusion is examined by increasing and decreasing the overall occlusion by 25%. The mixing behavior of the color bands for different relative occlusions is shown in Figure 15. With 75% occlusion, the fluid in the proximal region moves more efficiently, as more cyan fluid is dragged to the distal region. In contrast, with 125% occlusion, the cyan fluid appears to be trapped in the upper region, except for a small amount that moves with the ACW into the central region. On the other hand, the fluid at the distal stomach mixes much more efficiently with the 125% occlusion, as the colors distribute much more uniformly compared to the 75% one. The inner fluid layers (green, yellow, red, and blue), separate into two distinct parts in all cases after 100 s, with the separation being more pronounced in the higher occlusion cases. The level of mixing intensity is significantly stronger in the lower region for the higher occlusion case, whereas the area of strong mixing is larger in the lower occlusion case. The tangential advection caused by the ACW predominantly affects the 75% occlusion case, as the fluid moves parallel to the wall. In contrast, the 125% occlusion case exhibits a pronounced retropulsive motion. The large contraction near the pylorus directs the fluid back into the central region and results in more robust mixing. Overall, the higher occlusion provides better mixing, as shown in Figure 16. The curves start from 0% and reach 19%, 23%, and 29% for relative occlusions of 75%, 100%, and 125%, respectively, at 6 min. At 20 min, they are predicted to reach 58%, 68%, and 79%. As shown in Table 3, the $t_{1/2}$ values are 15.8, 12.2, and 8.9 min for the 75%, 100%, and 125% relative occlusions, respectively.

**FIGURE 17:**
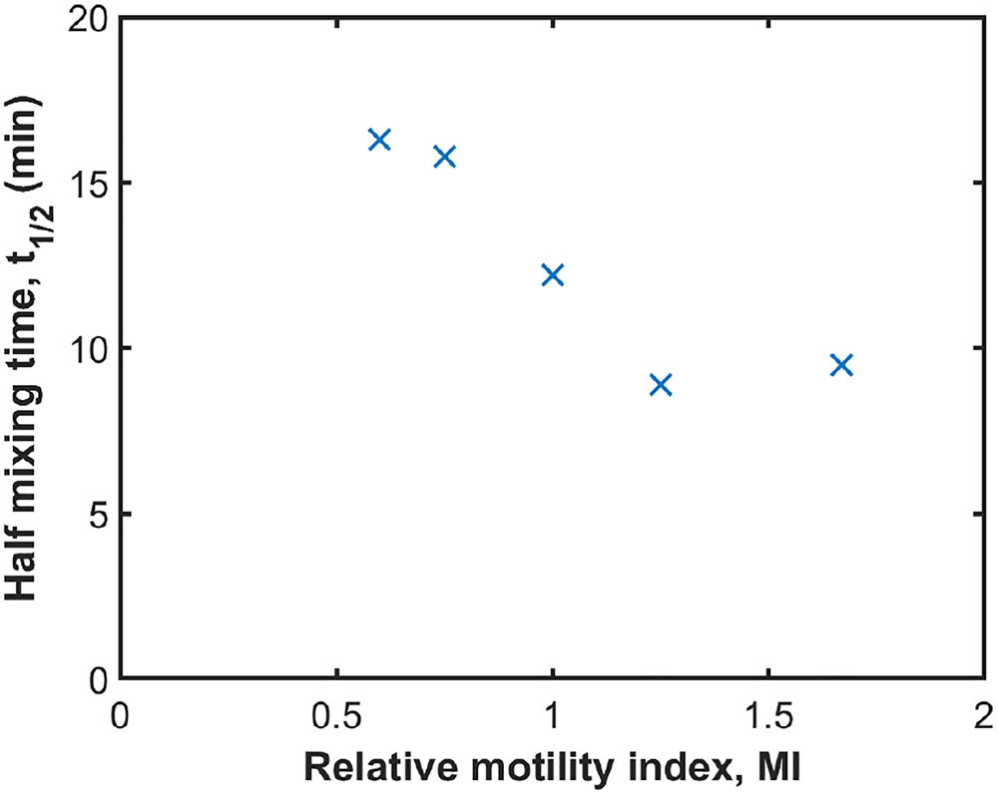
Effect of gastric motility on mixing performance.

The relationship between gastric motility and mixing performance is illustrated in Figure 17. Note that relative MI is calculated as the product of speed and occlusion. The $t_{1/2}$ generally decreases as MI increases, suggesting that enhanced gastric motility leads to more efficient mixing. Conversely, a reduced MI is commonly associated with gastroparesis, which is a condition characterized by impaired gastric motility. Results show that it leads to a larger $t_{1/2}$ thus slower mixing. However, the last two data points in Figure 17 exhibit a reverse trend. This outliner indicates more complex behaviors at high MI, for which further investigation is needed. It may imply that the relative occlusion should have a higher weighting than ACW speed, resulting in a smaller MI.

Figures S5–S8 show that the effects of contraction wave speed and relative occlusion on pH changes are not significant, as the acid‐rich layer thickness and pH changes remain nearly the same for the variations of speed and occlusion. These findings suggest that neither contraction wave speed nor relative occlusion significantly impact pH transport because these control the tangential fluid advection and contribute only weakly to radial advection, indicating that other factors, such as diffusion, play a more critical role.

### 4.3 Effect of acid diffusion coefficient

**FIGURE 18:**
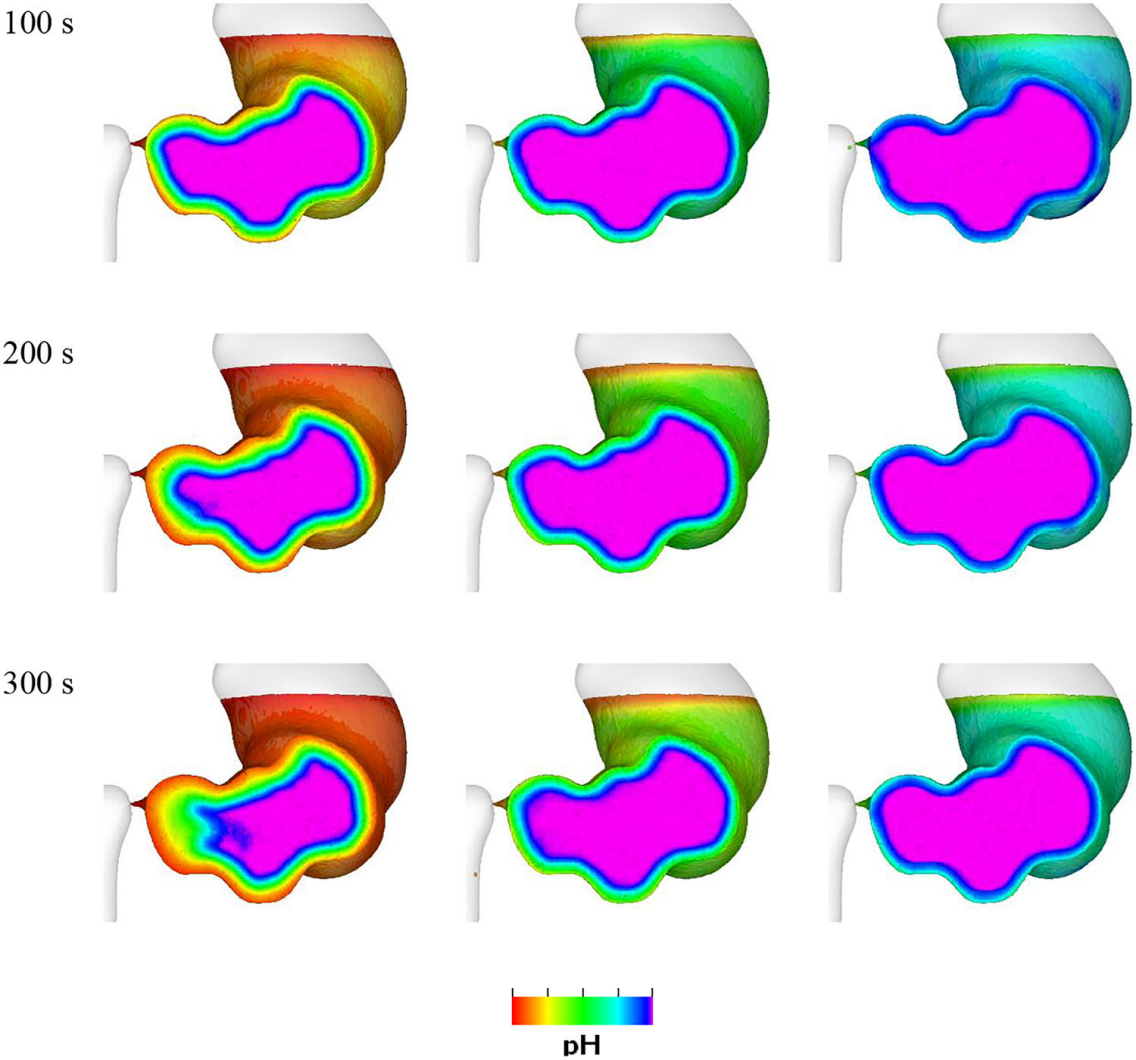
Visualization of pH changes in the fluid for different acid diffusion coefficients at 100 s, 200 s and 300 s. (left) 10^−10^ m^2^ s^−1^; (middle) 10^−9^ m^2^ s^−1^; (right) 10^−8^ m^2^ s^−1^.

The effect of the acid diffusion coefficient is investigated by comparing the results with diffusion coefficients of 10^−10^, 10^−9^, and 10^−8^ m^2^ s^−1^. The changing pH in the fluid over time with different diffusion coefficients is visualized in Figure 18. As expected, a higher diffusion coefficient leads to greater acid transport into the stomach. This means that the acid molecules move more quickly and efficiently through the fluid, resulting in a more rapid and extensive distribution of acid. Consequently, the overall acid concentration in the stomach is significantly higher.

**FIGURE 19:**
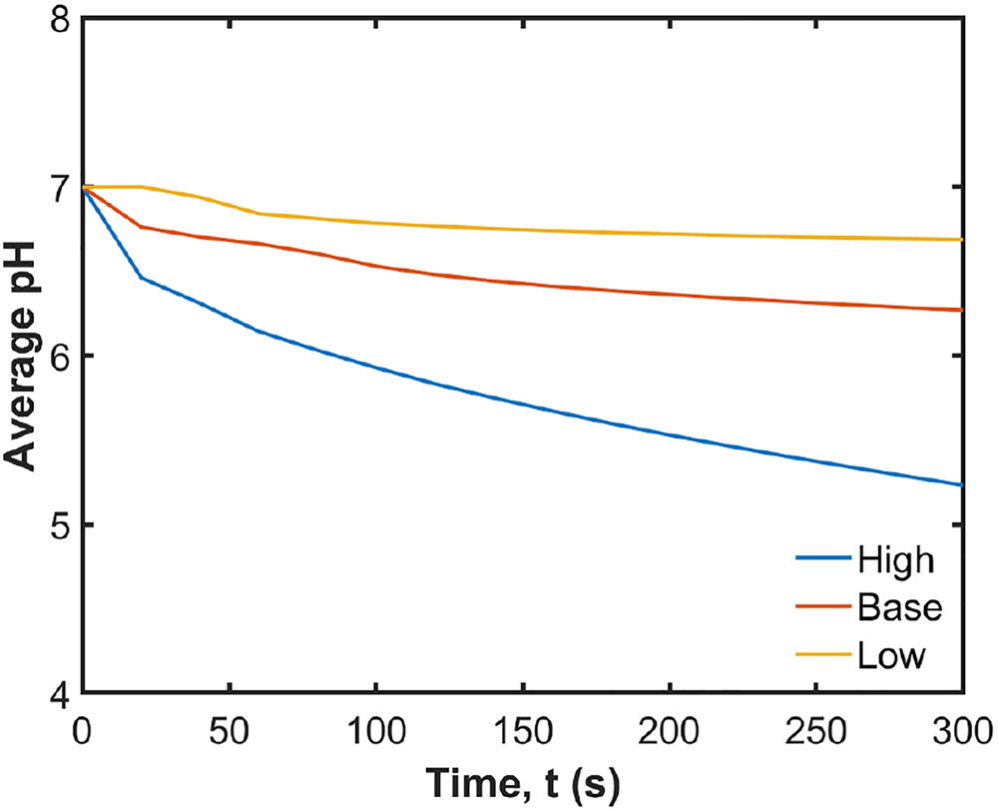
Effect of diffusion coefficient on average pH in the stomach.

The effect of the diffusion coefficients on the average pH is shown in Figure 19. The average pH decreases much more quickly with a higher diffusion coefficient. The average pH of the fluids starts from 7.0 and decreases to 6.7, 6.3, and 5.2 at 300 s, with acid diffusion coefficients of 10^−10^, 10^−9^, and 10^−8^ m^2^ s^−1^, respectively. These results are broadly consistent with in vitro observations, where pH values drop to 6.8 within 5 min during milk protein digestion (Wang et al. 2018) and to 5.8 during protein digestion (Mennah‐Govela et al. 2021). In this study, the secretion rate is dependent on the extent of contact between the internal fluid and the stomach wall and the corresponding acid secretion rates over the 5 min period are 0.008, 0.03, and 0.5 mL/min. Compared to the typical secretion rate of 1 mL/min during fasting (Mennah‐Govela et al. 2021), these rates are lower but still fall within a physiologically plausible range, especially given the simplified assumptions of the model.

**FIGURE 20:**
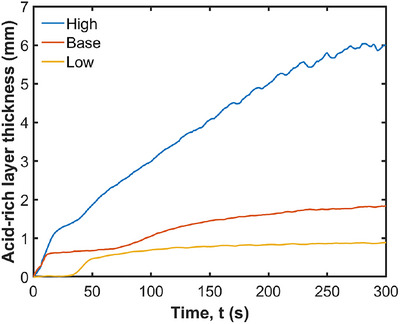
Effect of diffusion coefficient on acid‐rich layer thickness.

Fluids with higher acid diffusion coefficients will experience even more rapid and extensive acid mixing, potentially enhancing the overall digestion process. Figure 20 illustrates the effect of the diffusion coefficient on the acid‐rich layer thickness (of fluid with a pH below 6.8). This increases significantly with the increasing diffusion coefficient. After 5 min, the thickness of the acid‐rich layer varies significantly with the diffusion coefficient. For the low diffusion coefficient, the layer is only 1.1 mm thick. With the base diffusion coefficient, it increases to 2.2 mm, and for the high diffusion coefficient, it reaches 7.2 mm. Given that the stomach's geometry has a half‐diameter of 40 mm at the fundus, an acid‐rich layer thickness of 7.2 mm represents approximately 18% of the total fluid thickness. These findings indicate that a higher diffusion coefficient leads to a substantially thicker acid‐rich layer, suggesting that the rate of acid diffusion through the fluid is crucial for determining the extent of acid penetration and distribution. In contrast, the effect of the ACW on acid‐rich layer thickness is much less pronounced. While variations in ACW influence the mixing dynamics, they do not significantly enhance the transport of pH within the fluid. This indicates that the diffusion coefficient is the dominant factor in acid uptake, compared with the impact of physical mixing induced by the ACW. While Kozu et al. (2010) simulated pepsin instead of acid diffusion in their study, a related conclusion was drawn that the rate of diffusion may act as a limiting factor in food digestion within the stomach. At the early time points, the thicknesses of the acid‐rich layer have a spatial resolution of less than 1 mm as shown in Figure 20, which appears to be below the SPH particle resolution. This is justified through several considerations. First, the measurement is an average over the wetted surface, so values below the resolution are possible from the spatial averaging. Second, the acid‐rich thickness represents an indicative distance at which the acid concentration is at the pH of 6.8. However, the acid extends well beyond this point toward the middle of the stomach, with progressively decreasing concentrations (essentially resembling a one‐dimensional error function solution of the diffusion equation with distance). Accordingly, the actual solution to the diffusion equation spans several SPH particles, so the continuum solution by SPH is valid. When the acid‐rich layer thickness is over one single SPH particle, it captures the high‐concentration region of a wider solution that has sufficient resolution to predict the diffusion.

## 5 Conclusions and Future Work

In this study, we used a computational stomach model with the SPH approach to investigate the gastric mixing behavior and acid transport in the stomach. The extreme anisotropy of the advection generated by the ACW waves results in highly effective tangential flow along the walls, driving circulatory flow and mixing. This also causes the acid to accumulate at the top of the stomach contents. On the other hand, the weak transverse (radial) advection means there is little advective augmentation of the diffusion, which is dominantly radial. The overall mixing is less intense in the upper region and more robust in the lower region, characterized by the varying occlusions of the ACW in different areas. This observation is consistent with established gastric physiology, where the proximal (upper) stomach functions primarily as a reservoir for ingested food, while the distal (lower) stomach serves as a pump, facilitating mixing and propulsion.

Fluid viscosity significantly affects mixing dynamics, with higher viscosity fluids exhibiting slower and less efficient mixing. However, viscosity does not substantially impact acid ingress, due to poor radial advection of the acid transport. Both contraction wave speed and relative occlusion significantly enhance fluid mixing. Faster contraction waves and greater occlusion lead to more rapid mixing of gastric contents with significantly enhanced circulatory and retropulsive flow. However, these factors have a minimal impact on acid distribution, as they mainly influence tangential fluid advection and only weakly affect radial advection.

The acid diffusion coefficient is the dominant factor in determining pH changes within the stomach. A higher diffusion coefficient results in more rapid and extensive acid transport, leading to greater pH changes, faster the digestion process and nutrient absorption. In summary, our study highlights the complex interaction between mechanical and chemical digestion processes in the stomach.

Future work for this model will focus on several key areas to enhance its accuracy and comprehensiveness. An important aspect will be enabling the pylorus to open for discharge, allowing the study of both gastric mixing and emptying. Different acid diffusion coefficients based on specific food compositions should be employed, rather than the constant coefficient used in this study. Additionally, a comprehensive model would require integrating complex models of food breakdown and chyme chemistry. This includes simulating large, undigested food fragments with realistic microstructure and surface topology, along with modeling the mass diffusion of acid and other chemical species into the food matrix. The mechanisms of food breakdown vary significantly depending on the food type, yet only two studies have incorporated any aspect of this into their models. Skamniotis et al. (2020) modelled the disintegration of an oatcake bolus using a viscoplastic‐damage constitutive law calibrated through rheological experiments. However, their simulation excluded the surrounding fluid and focused on the breakdown of the bolus due to the wall deformation. More recently, Ghosh et al. (2025) simulated the breakdown of solid breads using elastic–plastic and elastic–brittle constitutive laws based on an experimental study conducted by Marciani et al. (2001). The integration of solid food breakdown into gastric models remains highly limited, largely due to the complexity of food structures and the lack of detailed experimental observations. Early future studies should focus on modeling food contents with simple measurable compositions (pure carbohydrates or proteins), accompanied by experimental validation to establish confidence in the model predictions.

## Nomenclature

C [kg/m^3^]: Mass concentration
$c_{s}$ [m/s]: Local speed of sound
$D_{m}$ [kg/m/s]: Mass‐based species diffusion coefficient (=ρD)
f [m/s^2^]: Acceleration due to fluid‐wall forces
$f_{mix}$ [‐]: Mixing efficiency
g [m/s^2^]: Acceleration due to gravity
h [m]: Smoothing length
m [kg]: Mass of a particle
P [Pa]: Pressure
r [m]: Particle position vector
t [s]: Time
U [m/s]: Characteristic fluid velocity
v [m/s]: Particle velocity
W [m^−3^]: Interpolation function
Δt [s]: Timestep
Δx [m]: Initial particle spacing
μ [Pa.s]: Dynamic viscosity
ρ [kg/m^3^]: Fluid density
Υ [‐]: Material constant applicable to fluids
η [‐]: Modelling parameter to regularise singularities
κ [‐]: Modelling constant in the viscous term
ξ [‐]: State of mixing
σ [‐]: Grid‐averaged value of standard deviation

## Author Contributions

**Xinying Liu**: methodology, data curation, formal analysis, investigation, conceptualization, visualization, writing – original draft, validation. **Simon M. Harrison**: conceptualization, methodology, software, writing – review and editing, resources, formal analysis, investigation, validation. **Shouryadipta Ghosh**: methodology, writing – review and editing, investigation. **David F. Fletcher**: conceptualization, investigation, project administration, funding acquisition, writing – review and editing, validation, supervision, methodology. **Paul W. Cleary**: conceptualization, investigation, writing – review and editing, methodology, software, project administration, resources, validation.

## Conflicts of Interest

The authors declare no conflicts of interest.

## Supporting information




**Supplementary Figures**: jfds70671‐sup‐0001‐figuresS1‐S8.docx
