# Supplementary material for: Gastric Mixing and Acid Diffusion in a Human Stomach Simulated Using Smoothed Particle Hydrodynamics
Source: J Food Sci. 2025 Nov 7;90(11):e70671. doi: 10.1111/1750-3841.70671 (PMC12595260; doi:10.1111/1750-3841.70671)
Supplement: Supplementary file 1 — Supplementary Figures: jfds70671‐sup‐0001‐figuresS1‐S8.docx [file JFDS-90-0-s001.docx]

**Supplementary material**


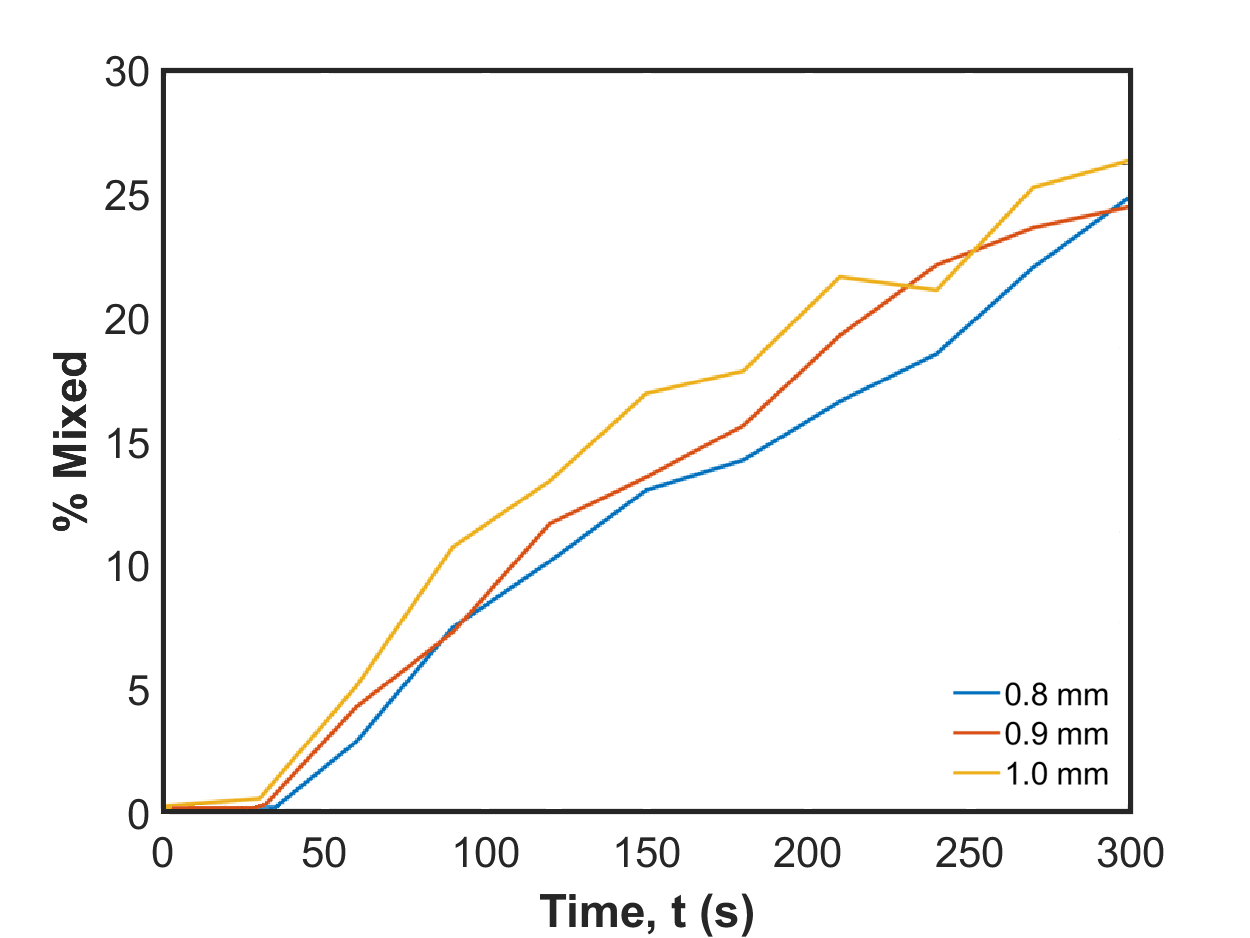


Figure S1: Effect of spatial resolution on mixing efficiency in the stomach.


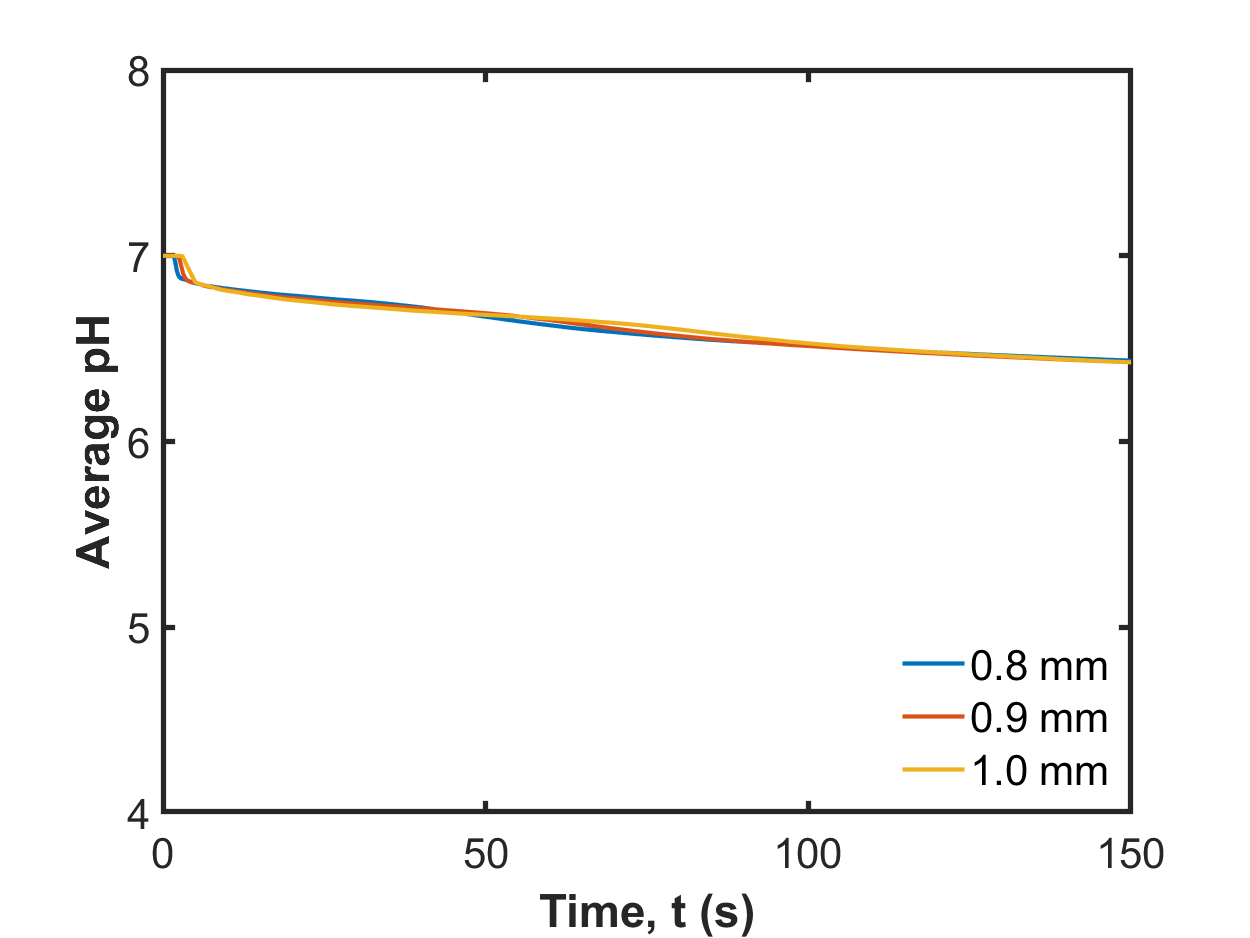


Figure S2: Effect of spatial resolution on average pH in the stomach.


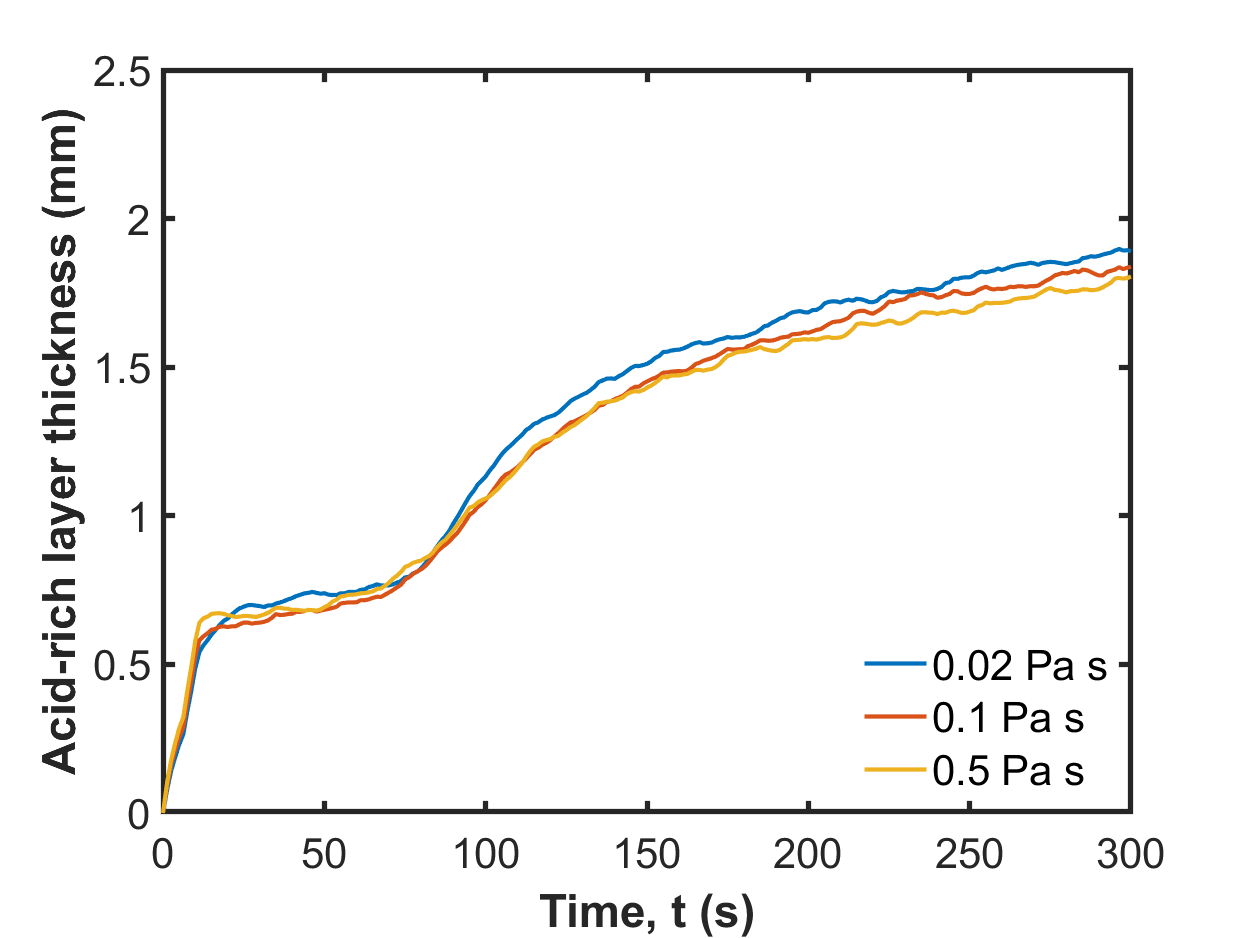


Figure S3: Effect of viscosity on acid-rich layer thickness.


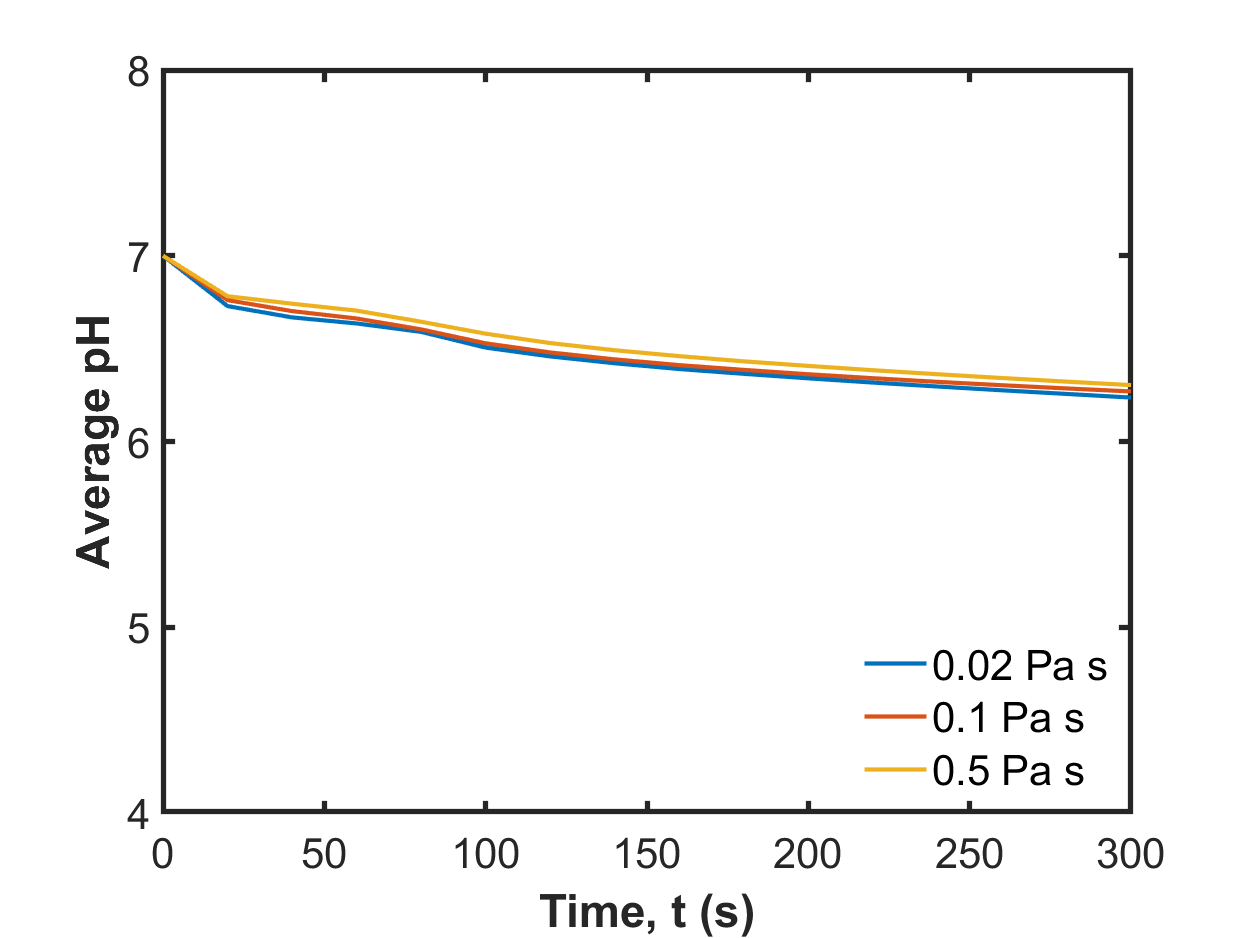


Figure S4: Effect of viscosity on pH changes.


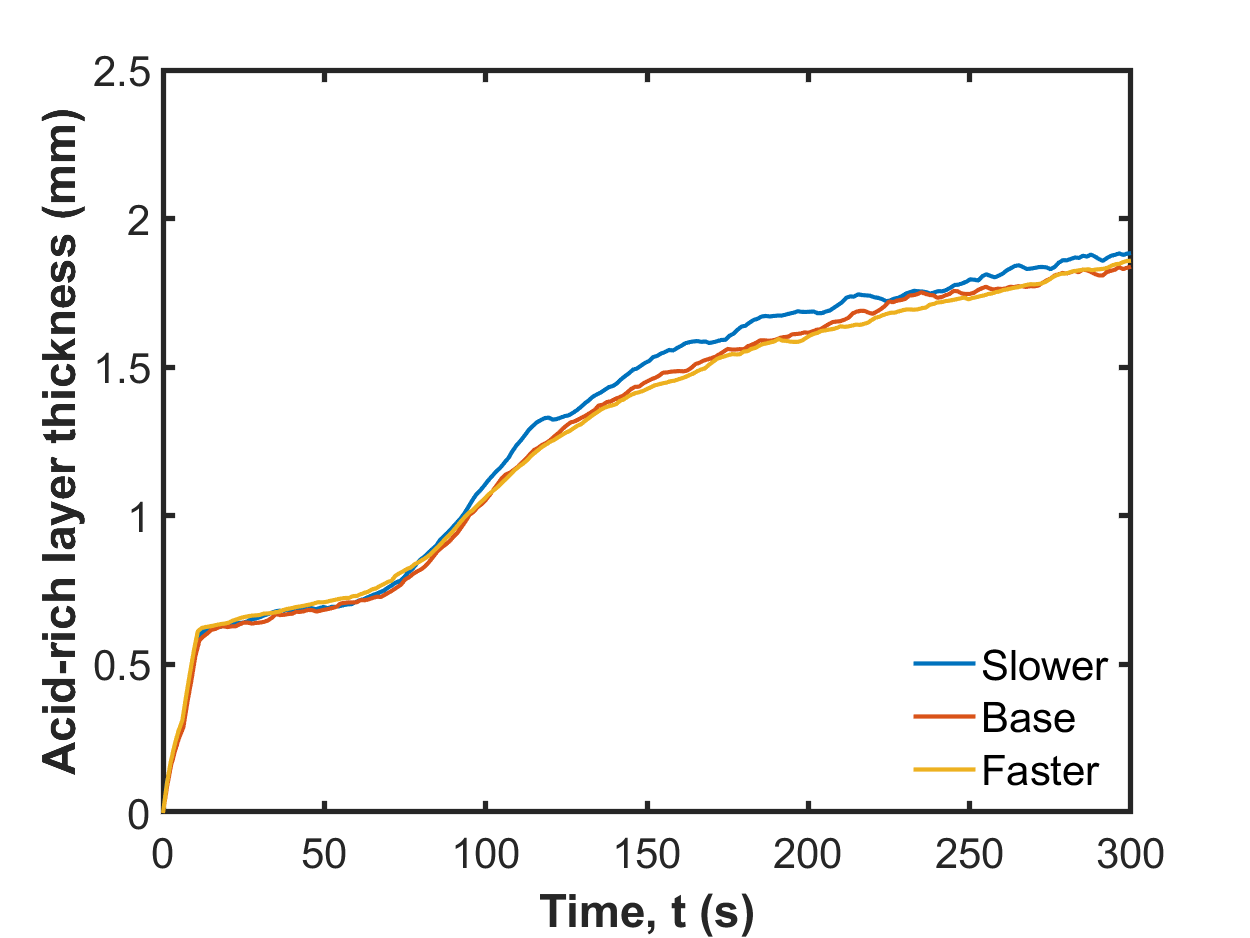


Figure S5: Effect of wave speed on acid-rich layer thickness.


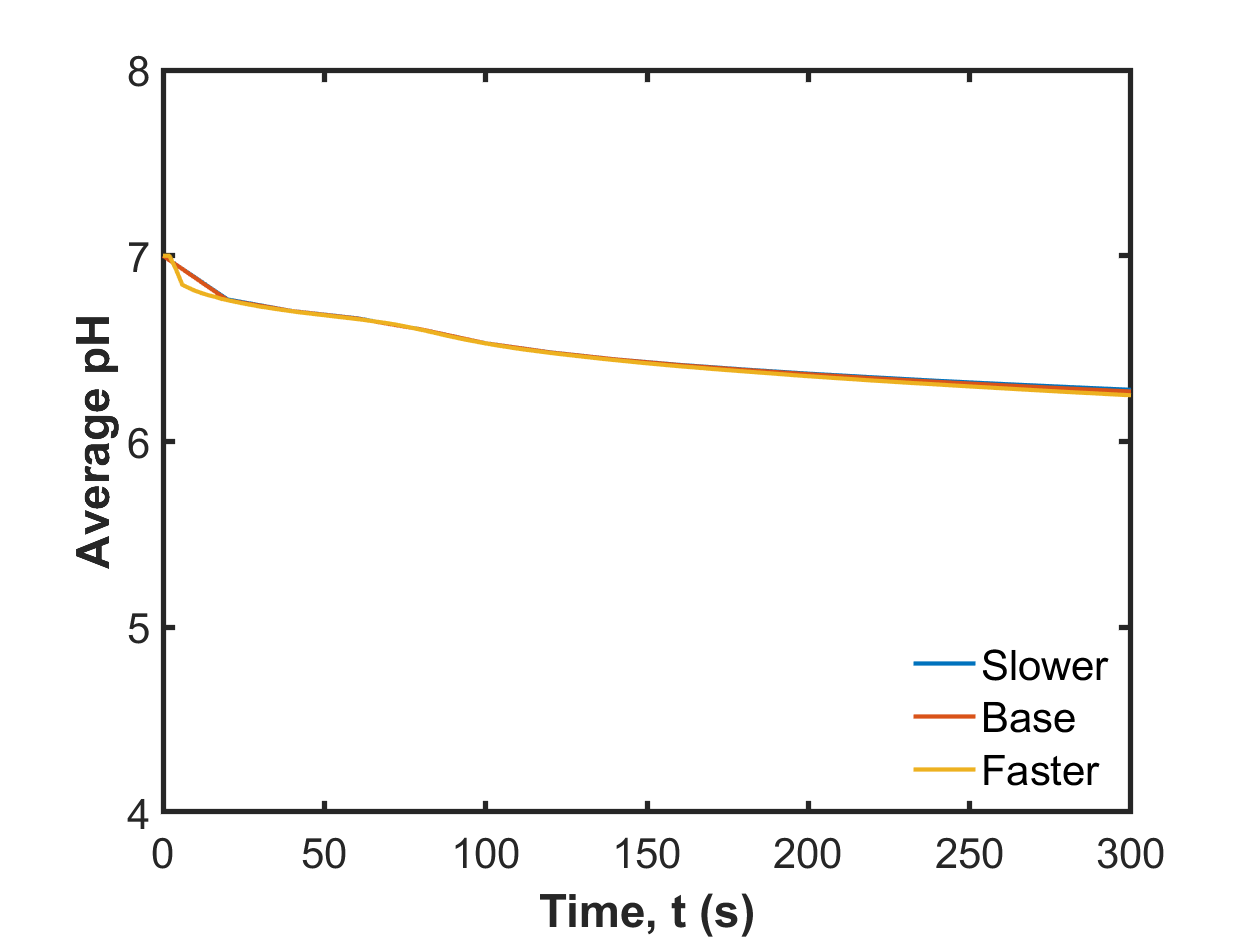


Figure S6: Effect of wave speed on pH changes.


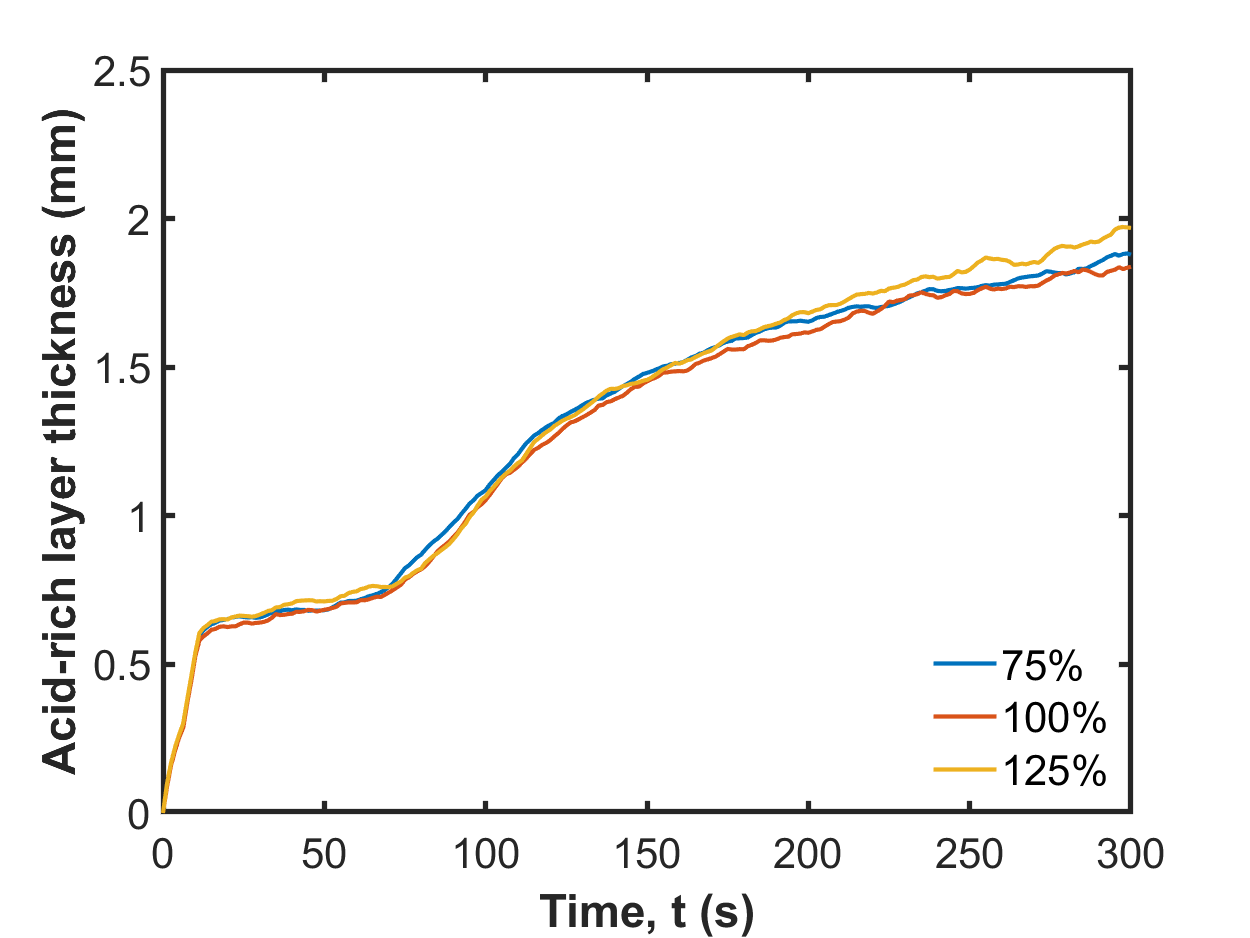


Figure S7: Effect of relative occlusion on acid-rich layer thickness.


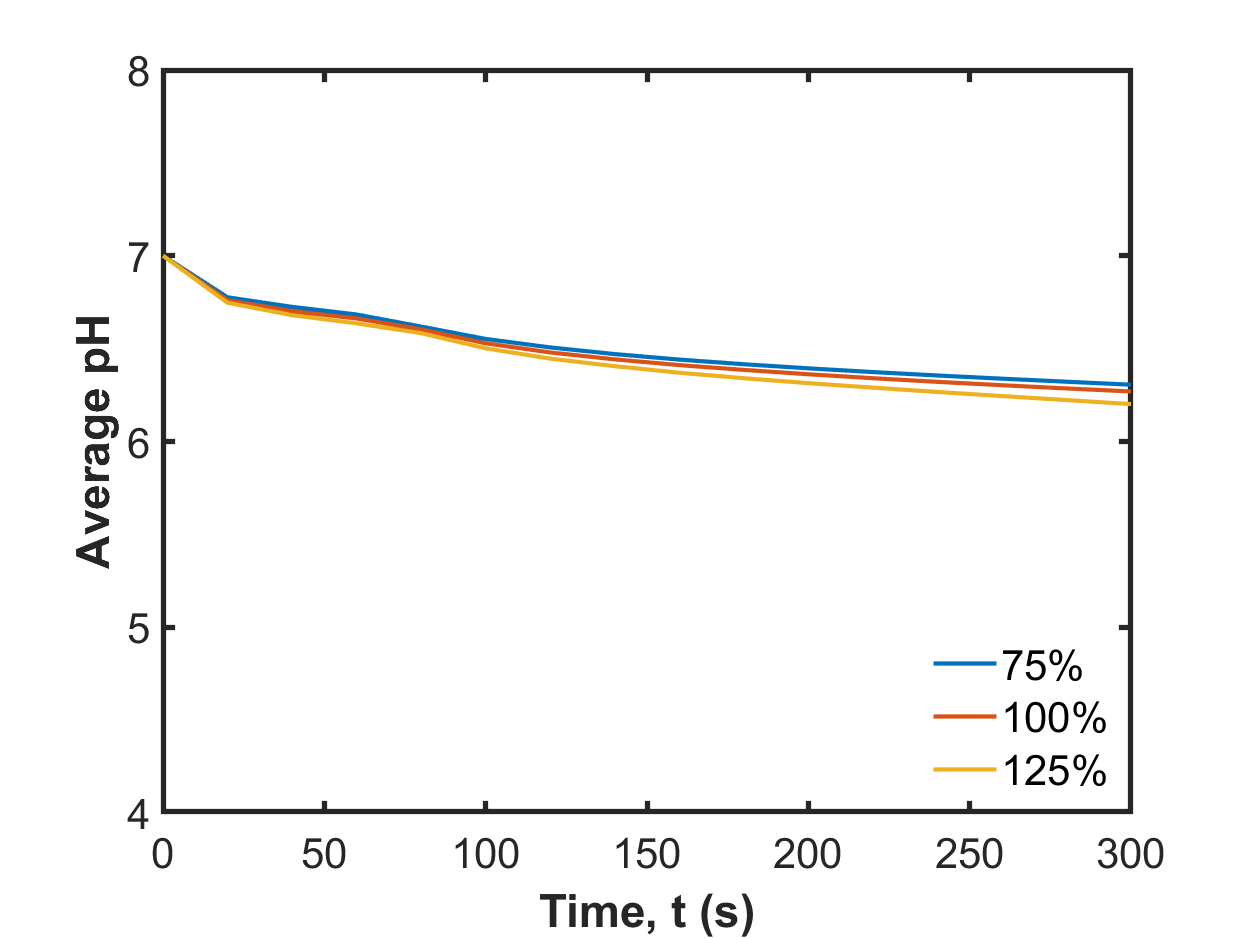


Figure S8: Effect of relative occlusion on pH changes.
